# Transcriptomic effects of rs4845604, an IBD and allergy-associated RORC variant, in stimulated ex vivo CD4+ T cells

**DOI:** 10.1371/journal.pone.0258316

**Published:** 2021-10-21

**Authors:** Paul A. Wilson, Sara Santos Franco, Liu He, Nicholas W. Galwey, Jackie Meakin, Rebecca McIntyre, Simon M. McHugh, Michael A. Nolan, Sarah L. Spain, Thaddeus Carlson, Mercedes Lobera, Justin P. Rubio, Bill Davis, Linda C. McCarthy

**Affiliations:** 1 Human Genetics, GlaxoSmithKline Medicine Research Centre, Stevenage, England; 2 Clinical Unit Cambridge, Addenbrooke’s Centre for Clinical Investigation, GlaxoSmithKline, Cambridge, England; 3 Wellcome Sanger Institute, Hinxton, Cambridge, United Kingdom; 4 Research Statistics, GlaxoSmithKline Medicines Research Centre, Stevenage, England; 5 Functional Genomics, GlaxoSmithKline Medicines Research Centre, Stevenage, England; 6 Quench Bio, Cambridge, MA, England; 7 Open Targets, Hinxton, Cambridge, England; 8 Adaptive Immunity, GSK Pharma Research & Development, Cambridge, MA, United States of America; 9 Department of Pharmacology and Therapeutics, The University of Melbourne, Victoria, Australia; 10 The Florey Institute of Neuroscience and Mental Health, The University of Melbourne, Victoria, Australia; University of California San Diego, UNITED STATES

## Abstract

RORγt is an isoform of RORC, preferentially expressed in Th17 cells, that functions as a critical regulator of type 3 immunity. As murine Th17-driven inflammatory disease models were greatly diminished in RORC knock-out mice, this receptor was prioritised as an attractive therapeutic target for the treatment of several autoimmune diseases. Human genetic studies indicate a significant contributory role for RORC in several human disease conditions. Furthermore, genome-wide association studies (GWAS) report a significant association between inflammatory bowel disease (IBD) and the RORC regulatory variant rs4845604. To investigate if the rs4845604 variant may affect CD4^+^ T cell differentiation events, naïve CD4^+^ T cells were isolated from eighteen healthy subjects homozygous for the rs4845604 minor (A) or major (G) allele). Isolated cells from each subject were differentiated into distinct T cell lineages by culturing in either T cell maintenance medium or Th17 driving medium conditions for six days in the presence of an RORC inverse agonist (to prevent constitutive receptor activity) or an inactive diastereomer (control). Our proof of concept study indicated that genotype had no significant effect on the mean number of naïve CD4 T cells isolated, nor the frequency of Th1-like and Th17-like cells following six days of culture in any of the four culture conditions. Analysis of the derived RNA-seq count data identified genotype-driven transcriptional effects in each of the four culture conditions. Subsequent pathway enrichment analysis of these profiles reported perturbation of metabolic signalling networks, with the potential to affect the cellular detoxification response. This investigation reveals that rs4845604 genotype is associated with transcriptional effects in CD4^+^ T cells that may perturb immune and metabolic pathways. Most significantly, the rs4845604 GG, IBD risk associated, genotype may be associated with a differential detoxification response. This observation justifies further investigation in a larger cohort of both healthy and IBD-affected individuals.

## Introduction

RORC is a ligand-dependent nuclear hormone receptor with validated functional roles in a wide variety of biological processes, including: cellular immunity, steroid and glucose metabolism, and the peripheral circadian rhythm [1,2]. Of particular interest is the RORγt (ROR-gamma-t, RORγ2, RORC2, ROR-gamma isoform b), isoform of RORC, as it is preferentially expressed in a number of lymphoid cells, including T helper Th17 cells, a subset of FOXP3^+^ T-regulatory cells and type 3 innate lymphoid cells (ILC3), and functions as a critical regulator of type 3 immunity [3–5]. More recently, studies reported that some cholesterol metabolites are likely to be endogenous ligands of RORγt [2,6]. These combined characteristics suggest that RORγt may, in part, link type 3 immunity with the metabolic system.

The observation that Th17-driven inflammatory disease models were greatly attenuated in RORC knock-out mice [7] positioned this receptor as an attractive therapeutic target for the treatment of several autoimmune diseases. Consequently, RORC inverse agonists that suppress Th17 differentiation were identified [8,9] and their therapeutic potential evaluated in human clinical trials [10].

In addition to a plethora of mouse functional studies, human genetic studies have provided a persuasive body of evidence that is indicative of a significant contributory role for RORC in several human disease conditions. Using a combination of whole exome sequencing and genome wide linkage methods, Okada *et*.*al*. [11] identified three unique RORC loss-of-function mutations in consanguineous families, with each mutation resulting in impaired T cell expression of IL-17A/F and IFN-g and manifesting as chronic candidiasis and mycobacterial disease. Using a nested case-control design, Newman *et*. *al*. [12] reported three 5’ flanking or intron 1 variants of RORC as significant contributors to the development of secondary lymphedema following breast cancer treatment: this observation implies a causative role for RORγt, as this RORC transcript is expressed exclusively in lymphoid cells and is reported to be critical for lymphoid organogenesis [13]. Furthermore, a meta-analysis of Crohns disease and Ulcerative colitis genome-wide association studies (GWAS) reported a significant association between inflammatory bowel disease (IBD) and the RORC intronic variant, rs4845604 [14]. This variant was also found to be associated with IBD by de Lange *et*.*al*. [15], which further heightened interest in the RORγt transcript, as it is preferentially expressed in immune cells that are reported to regulate intestinal homeostasis [3,13]. More recently, a phenome-wide association study (PheWAS) of rs4845604 with self-reported phenotypes revealed a protective effect for several allergic traits [16].

To better understand if and how the RORC rs4845604 variant affects CD4^+^ T cell differentiation events, we compared both the cellular differentiation and transcriptional response of purified naïve CD4^+^ T cells *ex vivo* from nine subjects homozygous for the rs4845604 A allele (a protective IBD association), with nine subjects homozygous for the G allele (a risk allele for IBD). Genotype specific effects on T cell differentiation were studied by culturing isolated naïve T cells in the presence and absence of a cytokine cocktail optimised to drive TH17 production (see the S1 supplementary materials pre-study protocol optimisation section in S1 File), and both differentiation conditions tested in the presence of the RORC inverse agonist GSK2794778A (to prevent constitutive receptor activity), or an inactive diastereomer GSK2794776A (control compound). Our analysis identified rs4845604 variant-associated effects on gene expression that may perturb inflammatory and metabolic signalling pathways previously associated with human disease.

## Materials and methods

### Recruitment and genotyping

Ethical approval (LREC ref: 08/H0302/100, UK) and written informed consent were obtained prior to conducting this study. Briefly, eighteen genetically stratified, healthy volunteers were recruited from a bioresource maintained at GSK’s Clinical Unit in Cambridge, UK (https://volunteers.gsk.co.uk/): nine subjects were homozygous for the major (G) rs4845604 allele and nine homozygous for the minor (A) rs4845604 allele. Note that the bioresource archives DNA extracted from peripheral venous blood samples from volunteers who had given consent to be (1) genotyped at any gene of interest, and (2) re-contacted if their genetic status makes them potentially eligible to participate in further studies. The subjects used to complete this investigation were recruited solely on the basis of their rs4845604 genotype, although genotypes at rs11209026, rs34536443, and rs10758669 of subjects were also considered during analysis. Genotyping for rs4845604, rs11209026, rs34536443, and rs10758669 was completed using the Affymetrix AxiomTM Biobank Plus GSK Custom array. Additionally, for rs4845604, Fluidigm assays were carried out using 192.24 IFC (cat no 103–6338) and SNP Type Assays as described in the manufacturer’s instructions. Specific Target Amplification (STA) primers were also used for pre-amplification, as described in the SNPtrace Panel user guide pages 24–25. Custom primers for the rs4845604 Fluidigm assay were designed on the forward strand and ordered via Fluidigm (catalogue number ASY-GT-S). The primer sequences used were as follows: ASP1:GTTCCCTTTTCCTTTGTTCTCTCCTA, ASP2: TCCCTTTTCCTTTGTTCTCTCCTG, LSP:GCCTGAAGCATGAAAGGAAACACT, STA: GCCTTTTTTCACAAGGCGG

### Isolation of Naïve T Cells from Human Whole Blood

Approximately 150 mL of whole blood from each subject was collected on sodium heparin tubes (BD #368480) and the PBMCs isolated by Ficoll gradient. Briefly, whole blood was centrifuged in accuspin tubes (Sigma #A2055-10EA) containing 15 mL of Ficoll (GE Healthcare #17-1440-03) at 800 xg for 20 minutes at room temperature (RT). Avoiding the RBCs, the PBMCs were transferred to a tube containing 1 X DPBS (Gibco #14190–169) and centrifuged for 15 minutes at 300 xg (RT). The supernatant was discarded, and the pellet resuspended in 1 X DPBS for cell counting using a particle counter (Beckman Coulter). After passing the cell suspension through a 70 μm cell strainer, cells were centrifuged at 300 xg for 10 minutes (RT) and the pellet resuspended in separation buffer [1 X PBS (Gibco #10010–015), 0.5% BSA (Sigma #A7409) and 2mM EDTA (Life Technologies #15775–038), pH 7.2] in preparation for magnetic separation. Isolation of highly purified naïve CD4^+^ T cells was achieved by depletion of magnetic labelled cells as described by the manufacturer (Miltenyi Biotec #130-094-131). Briefly, after removing completely the supernatant, the pellet was resuspended in separation buffer (4 μL/ 1 x 10^6^ cells) and naïve CD4^+^ T-cell biotin-antibody solution (1 μL/ 1 x 10^6^ cells). Cells were incubated for 6 minutes at 2–8°C and then separation buffer was added (3 μL/ 1 x 10^6^ cells) together with Miltenyi bead solution (2 μL/ 1 x 10^6^ cells). After 11 minutes of incubation at 2–8°C, 3 mL of separation buffer was added to the cells. MACS columns (Miltenyi Biotec #130-042-401) were mounted on a magnetic separator and washed with 3 mL of separation buffer, before addition of the 3 mL of cell suspension. The cells flowing through the column (highly enriched in naïve CD4^+^ T cells) were collected, and the suspension was diluted in warm T-cell medium [10% heat inactivated FBS (Gibco #10500–064), 1 X GlutaMAX, and 1 X penicillin-streptomycin in RPMI 1640 (LifeTechnologies #31870–074)] for cell counting.

### Cell culture of Naïve T cells and Th17 stimulation

After centrifuging the cell suspension for 10 minutes at 300 xg (RT), each cell pellet was resuspended in T-cell medium (2 x 10^6^ cells/mL) and the cultures rested in a 5% CO_2_ and 37°C incubator for 90 minutes. Four culture conditions were created per subject: in the presence of either GSK2794776A or GSK2794778A [17] and in Th17 driving medium [T-cell medium containing 10 ng/mL IL-1β (Cell Guidance Systems #GFH167AF), 20 ng/mL IL-6 (BioVision #6464–10), 10 ng/mL IL-23 (BioVision #6470–10), 2 ng/mL TGFβ (BioVision #6479–10), 1 ng/mL IL-2 (BioVision #6461–10), 2 μg/mL anti-IL-4 (BioLegend #500815), 2 μg/mL anti-IFNγ (BioLegend #506513)] or in T-cell maintenance medium. Isolated naïve CD4^+^ T cells were then distributed into a 6-well plate containing each of the four conditions stated above in a cell density of 1 x 10^6^ cells/mL. Simultaneously, Dynabeads Human T-Activator CD3/CD28 (Life Technologies #11131D) were prepared according to the manufacturer instructions and resuspended in T cell medium. Bead suspension was then added to each well to give a final ratio of 1 bead: 50 cells, as described by Purvis et al (2010) [18]. Cells were incubated for 6 days in a 5% CO_2_ and 37°C.

Significant differences between groups were determined using unpaired t-tests. Analyses were performed using Microsoft Excel and a p-value < 0.05 was considered statistically significant.

### Flow cytometry

To assess changes in the percentage of naïve CD4^+^ T cells and Th1 and Th17 cells, at days 0 and/or 6 of culture, we used flow cytometry to analyse PBMCs, CD4^+^ cells and naïve CD4^+^ T-cells using blood extracted from non-genotyped, healthy subjects. that these subjects were not included in the main study, which focussed only on the genotyped subjects. Cells were stained for CD4 PerCP-Cy5.5 (BD #560650), CCR6 (CD196) PeCy7 (BioLegend #353418), CXCR3 (CD183) FITC (R&D #FAB160F), CD45RA APC-H7 (BD #560674), and CD3 V450 (BD #560365) for 30 minutes in the dark at room temperature. After staining, cells were washed in FACS Buffer (1 X PBS/1% FCS/0.1% NaN3) and analysed in a BD FACS Canto II flow cytometer using BD FACSDiva software (BD Bioscience). Compensation was performed on the BD FACS Canto II flow cytometer at the beginning of each experiment. Data was analysed using FlowJo v10.

To complete the primary study, FACS analysis were performed at days 0 and 6 of culture to characterize changes in cell populations of bloods extracted from genotyped subjects from the pilot phase (n = 6). Cells were harvested at day 6 of culture and stained for Live/Dead Aqua (Invitrogen #L34957), CCR6 PE (BioLegend #353410 and #353409), CXCR3 APC (BD #561732), and CD4 BV421 (BioLegend #317434) for 45 minutes on ice. After staining, cells were washed in PBS (Gibco #10010) and data acquired with a FACS Aria flow cytometer using BD FACSDiva software (BD Bioscience). Data was analysed using FlowJo v10.

### Preparation of cell pellets for mRNA analysis

After 6 days of cell culture (140 ± 6 hours), each culture well was mixed and transferred into a tube where the cells were counted after dilution in 1 X PBS (Gibco #10010–015). After centrifugation of the cell suspension at 500 xg for 6 minutes (RT), all supernatant was removed using a vacuum pump and the cell pellet immediately frozen on dry ice and stored at -80°C until RT-PCR or RNA-seq analysis. Cells were initially analysed for RORC and RORγt expression by RT-PCR at days 0 (baseline, before treatment), 1, 3, and 6, in order to choose the proper timepoint for RNA-seq analysis.

### Preparation of cell pellets for RT-PCR

Cells were prepared as described above and mRNA collected at days 0 (baseline, before treatment), 1, 3, and 6 for RT-PCR to quantify expression of RORC and RORγt and use these values to determine an optimum timepoint for RNA-seq analysis. RNA was extracted using Qiagen RNEasy kit no 74104 using the manufacturer’s instructions. Briefly, purified cells were resuspended in RLT buffer containing 10ul Beta Mercaptoethanol per ml, then vortexed and transferred to a Qiashredder tube (cat no 79654) before centrifugation at 12,000rpm for 2 mins. An equal amount of 70% ethanol was added to the eluate and mixed by pipetting, then transferred and bound by centrifugation to an Rneasy mini-column. The columns were rinsed by centrifugation with RW1 buffer and then on column DNAse digestion carried out. Columns were further rinsed with RPE buffer containing ethanol and then dried by centrifugation. RNA was double-eluted in 30ul RNAse/DNAse free water. Quantification was measured using a Nanodrop according to manufacturer’s instructions and quality assessed on an Agilent RNA Tapestation.

First strand cDNA was synthesised from 500ng of each RNA sample using ABI Hi Capacity cDNA kit (Life Technologies no 4368813), primed with random hexamers. Triplicate RT reactions were performed along with an additional reaction in which the reverse transcriptase enzyme was omitted to allow for assessment of genomic DNA contamination in each sample, then incubated for 10mins at 25°C, 1hr at 37°C, 5mins at 85°C and then stored at 4°C until used.

The resulting cDNA products were divided into 25ng aliquots using a Mosquito HV robot (TTP labs, Melbourne, Cambs) for parallel TaqMan PCR reactions using different primer and probe sets for quantification of multiple cDNA sequences.

TaqMan PCR was carried out using an ABI QuantStudio 12K Flex Real-time qPCR system (ThermoFisher) on the cDNA samples; 1x TaqMan Gene Expression Master Mix (Life Technologies no 4369016), 0.9 μM each primer (Sigma-Aldrich), 0.1 μM TaqMan probe (Life Technologies), or 1x Assay-on-demand (Life Technologies) 50°C for 2 min, 95°C for 10 min followed by 40 cycles of 95°C for 15 s, 60°C for 1 min. A TaqMan primer and probe set for RORγt was designed from the GenBank sequence NM_001001523.1 using Primer Express software (Applied Biosystems).

Forward primer: 5’-GAAGGACAGGGAGCCAAGGC-3’;

Reverse primer: 5’-CTTGTCCCCACAGATTTTGCA-3’

TaqMan probe: 5’-6FAM-TCAGTCATGAGAACACAAATTGAAGTGATCCC-3’-TAM

RORC (variant 1)–Assay-on-demand Hs01076120_g1 (Life Technologies).

GAPDH–Assay-on-demand Hs0278991_g1 (Life Technologies).

PPIA–Endogenous control 4333763F (LifeTechnologies).

Assays-on-demand sequence information is proprietary and is not disclosed by LifeTechnologies. The CT’s were transformed to copy number, assuming 95% efficiency of the assay and adjusted to normalise for cDNA input. “No RT” values were subtracted from each replicate. All values were log 10 transformed and normalised by division of a housekeeper copy number (calculated using the same method). Group means, and standard error of the mean were calculated from individual values.

### RNA-seq

Detailed protocols are available at https://www.ebi.ac.uk/ega/studies/EGAS00001001590. Briefly, mRNA was isolated as described above and sequence libraries prepared from 100 ng total RNA using the Illumina TruSeq SBS Kit v3—HS (200-cycles). Libraries were sequenced on an Illumina Hi-Seq 2000 using paired‐end runs. QC metrics were collated using FastQC (https://www.bioinformatics.babraham.ac.uk/projects/fastqc/): one sample was discarded due to below-threshold cut-offs with respect to several features. Trimmed high quality (phred score >20) bases were mapped to the human genome build GRCh38.p5 using STAR [19] RNA-seq aligner (version 2.5.1) and the featureCount summarisation program [20] used to count transcript reads.

### Differential transcript expression analysis

All data manipulations were made using R version 3.4.1 (http://www.R-project.org.). A DGEList object was created from a table of the count data and rows of very low transcript counts removed using the filterByExpr() function included in the edgeR package version 3.20.9 [21]. Data were prepared for linear modelling using the voom package [22]. Correlation between samples originating from the same subject was controlled for using the limma [23] duplicateCorrelation() function. The design matrix included columns representing factors specifying the treatment, batch and gender variables and their respective interactions. A full-rank model was fitted, and the coefficients used as input to the eBayes() function to obtain estimates of differential expression between subjects. Four statistical contrasts were calculated. These were

1. Contrast_1: transcript counts derived from purified cells isolated from GG subjects, unstimulated and in the presence of the inactive diastereomer, relative to transcript counts derived from purified cells isolated from AA subjects, also unstimulated and in the presence of the inactive diastereomer.
2. Contrast_2: transcript counts derived from purified cells isolated from GG subjects, unstimulated and in the presence of the RORC inverse agonist, relative to those derived from AA subjects in the same conditions.
3. Contrast_3: transcript counts derived from purified cells isolated from GG subjects, TH17 stimulated in the presence of the inactive diastereomer, relative to those derived from AA subjects in the same conditions.
4. Contrast_4: transcript counts derived from purified cells isolated from GG subjects, TH17 stimulated in the presence of the RORC inverse agonist, relative to those derived from AA subjects in the same conditions.

The top ranked genes from each of the four contrasts were extracted using the topTable() function using a combined threshold of > 2-fold log ratio with an associated p-value < 0.01.

Pathway enrichment and predicted upstream regulatory event analyses of the respective differential expression profiles were completed using Qiagen’s Ingenuity Pathway Analysis suite [24]

### Power calculations

All power calculations were performed using the software PASS (NCSS, LLC, Version 12.0.2). The variance of cell counts was estimated on the basis of the number of naïve CD4+ T cells isolated from PBMCs from the 18 subjects recruited for this study (see S1 supplementary materials, Table 1 in S1 File). These values indicated that this study was powered to 74% and 98% to detect a 2-fold and 3-fold difference in cell number, respectively.

To determine the power of the differential expression analysis to detect genotype effects, we estimated the between-subject and within-subject variance components of two Th17 signature gene [17,25] transcripts by fitting the following mixed model:

- Response variable: log2(CPM) for each transcript
- Fixed-effect terms: compound*treatment + genotype + gender + batch
- Random-effect terms: subject + residual

The coefficient of variation (CV) for each response variable was then calculated as loge(2) × sqrt(var[subject] + var[residual]/n)where n = number of observations per subject = 4.

These analyses indicated that if CV = 19% (*i*.*e*. the value obtained for IL23A-001) the experiment would have 100% power to detect a 2-fold change in expression, 99% power to detect a 1.5-fold change, and 17% power to detect a 1.1-fold change. If CV = 111% (*i*.*e*. the value observed for IL17F-001) the corresponding values are 33%, 15% and 6% power respectively.

## Results

Healthy volunteers previously recruited to the Clinical Unit Cambridge (CUC) database were genotyped for rs4845604 (Chr1: 151801680), and nine subjects homozygous for the minor (A) allele were age and gender matched with nine subjects homozygous for the major (G) allele. Naïve CD4^+^ T cells were purified from peripheral blood mononuclear cells (PBMCs) of each subject, and an unpaired t-test used to compare the baseline population means (of the naïve CD4+ T cells normalised to the number of PBMCs) of each genotype: see the Pre-study protocol optimisation section in S1 supplementary materials for further details in S1 File. The observed difference in mean cell-number of the two genotypes (7.63 ± 4.07 for AA versus 8.15 ± 3.83 for GG) was not statistically significant (*i*.*e*. a p-value > 0.05), suggesting that genotype had no discernible effect on the naïve CD4^+^ T baseline cell number in healthy subjects (see S1 supplementary materials, Table 1 in S1 File). Note that a power calculation estimated that this study was powered to 74% and 98% to detect a 2-fold and 3-fold difference in cell number (respectively) and that smaller genotype effects on cell number would be unlikely to be detected.

### Flow cytometry analysis

**Fig 1:**
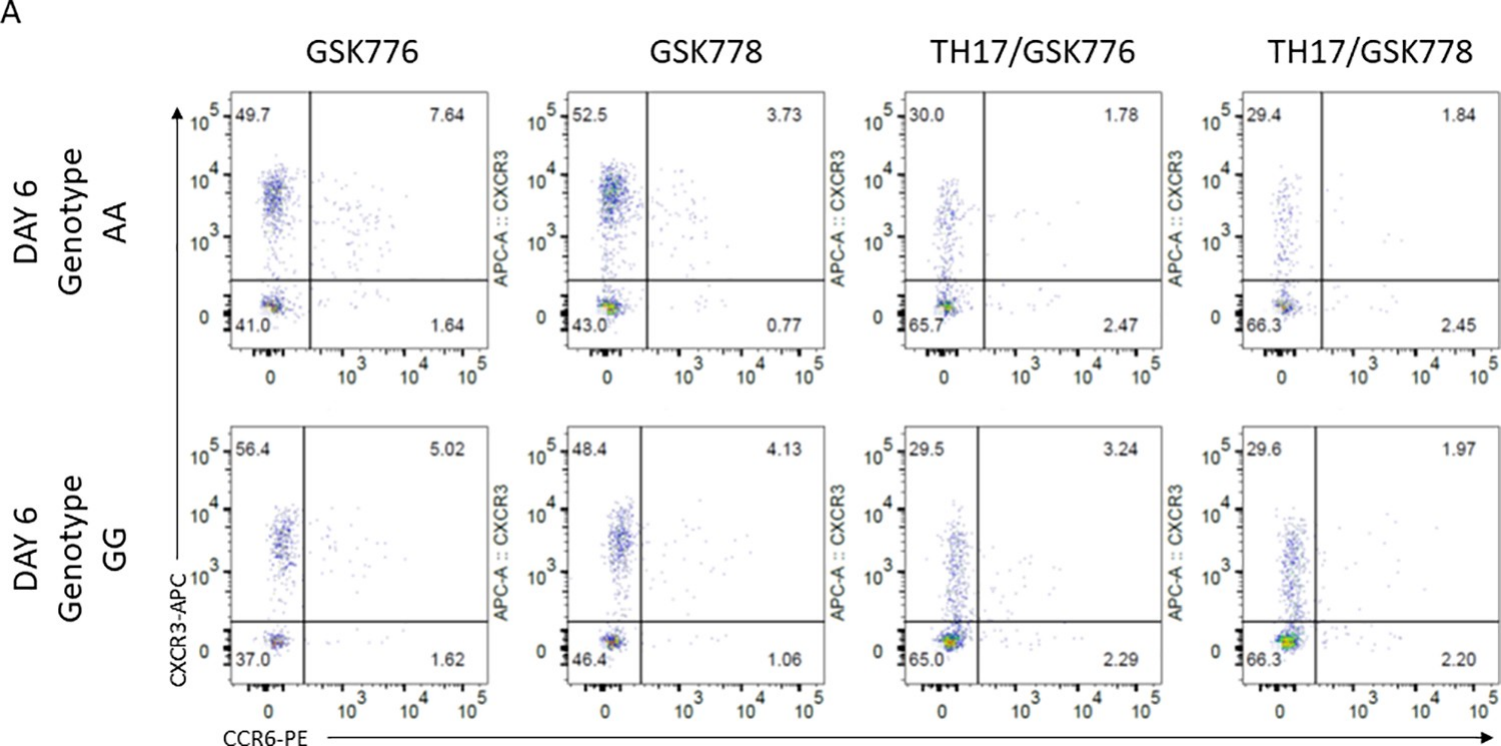
Flow cytometric analysis of CXCR3 versus CCR6 of live CD4^+^ cells at day 6 of culture was used to characterize the Th1-like and Th17-like cell populations. Representative graphical plots of cell count data from a subject of genotype group GG and genotype AA are shown, for each of the four culture conditions.

**Fig 2:**
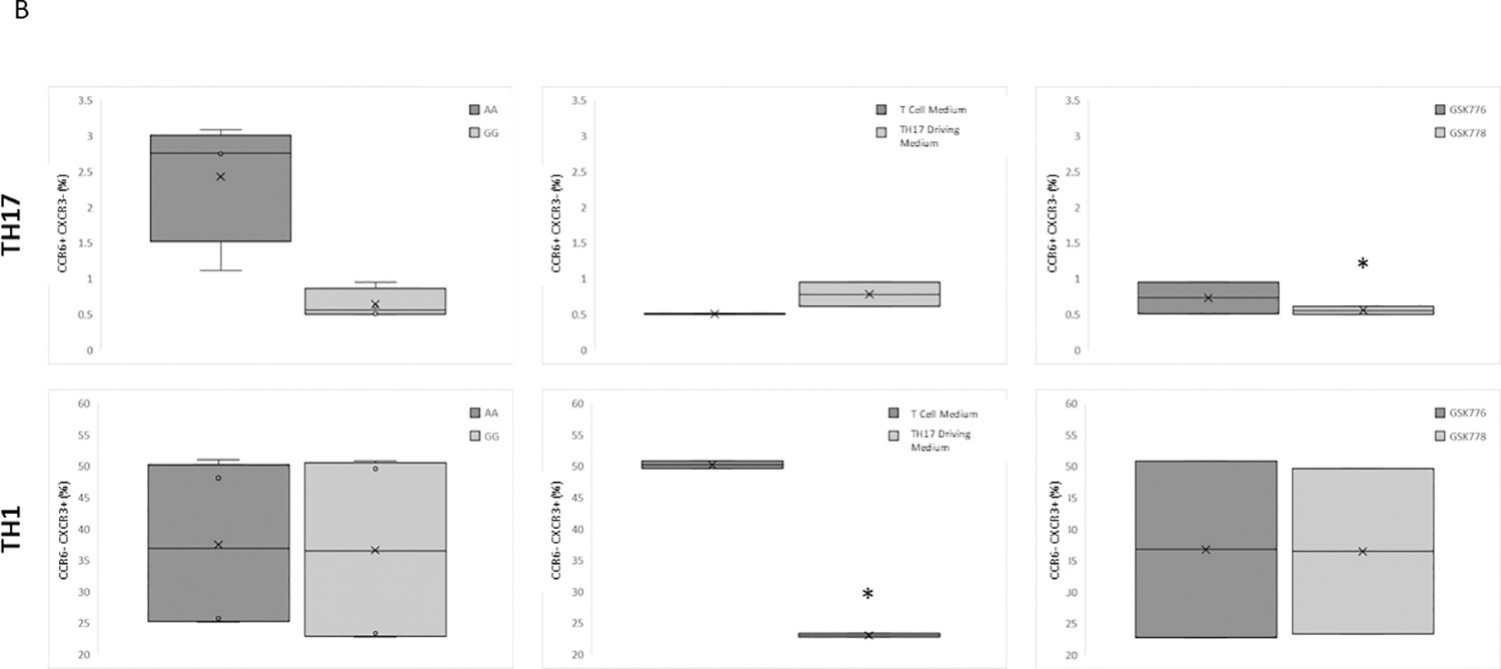
Characterization of the Th cell populations by flow cytometry at day 6 of cell culture under each of the four growth medium formulations: T cell maintenance medium or Th17 driving medium, in the presence of either GSK776 (GSK2794776A an inactive diastereomer) or GSK778 (GSK2794778A an inverse agonist) (n = 6, comprised of 3 subjects from each genotype group). Box and whisker plots representing the variation of Th1-like and Th17-like cell populations as a response to genotype, growth conditions, and compound treatment (i.e. left-to-right). The observed data indicate Th17 driven medium lead to a significant decrease of the Th1-like population and GSK2794778A to a decrease in the Th17-like population (unadjusted p-values < 0.05, are indicated with an *).

To assess the differentiation responses of naïve CD4+ T cells and determine the most suitable timepoints for sampling in the main expression study, cells from six genotyped subjects (3 subjects from each genotype group) were analysed using flow cytometry. T cell subtypes were classified as Th1-like [CXCR3+CCR6- from live CD4+ cells] and Th17-like [CXCR3-CCR6+ from live CD4+ cells] cells, following 6 days of culture. These extracellular markers are commonly used as identifiers of TH1-like and TH17-like phenotype, and have been shown to elucidate very similar subsets to those staining for IFNg and IL-17 (respectively) following stimulation and secretion block [26,27]. Cultures were classified with respect to genotype, active versus inactive compound, and culture condition (Figs 1 and 2). A mixed model, with subject and genotype each included as a random effect, and medium and compound included as fixed effects, was fitted to each of the response variables (i.e. Th1-like (%) and Th17-like (%)) to determine cell response to each of the four conditions. The ANOVA (Fixed Effect Tests) for Th17-like (%) reported a significant effect on Th17-like number dependent on the type of compound used (p-value = 0.03), but no significant effect with any other factor, or the interactions between these factors. The ANOVA for Th1-like (%) reported a highly significant effect on Th1-like numbers dependent on the medium used (p-value < 0.0001), but no significant effect for any other factor, or the interactions between the factors (S1 supplementary materials, Fig 5A in S1 File).

Taken together, the observed changes in Th1-like and Th17-like cell numbers indicated that incubation for six days in maintenance medium resulted in a significant increase in the percentage of Th1-like cells, while addition of the inverse agonist (GSK2794778A) reduced the percentage of Th17-like cells retrieved, and that genotype had no statistically significant effect on the number of either cell type in any of the four culture conditions.

### Cell proliferation and response to treatment

**Fig 3:**
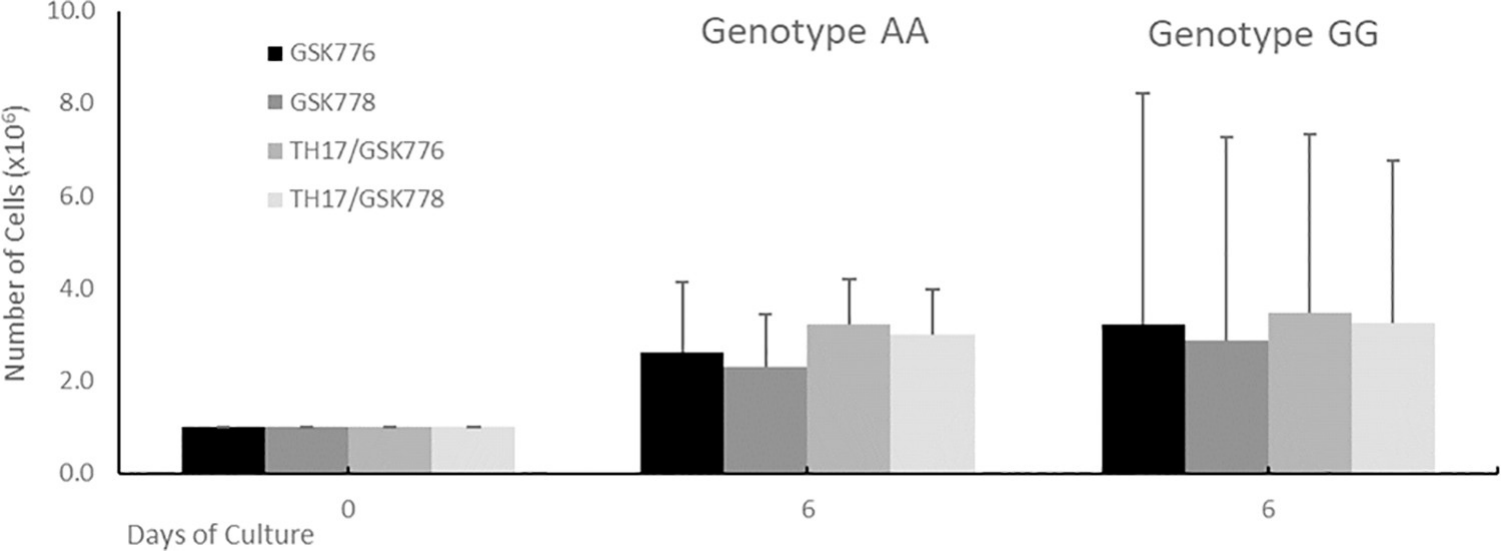
Genotype has no significant effect on CD4+ T cell proliferation. Cell proliferation outcomes in naïve CD4+ T cell counts following 6 days of culture, for each of the two genotypes under the four treatment conditions (i.e. Th17 driving medium or T cell maintenance medium in the presence of either GSK776 (GSK2794776A - an inactive diastereomer) or GSK778 (GSK2794778A -an inverse agonist of RORC)). Note that the cell number represents the average cell count derived from nine homozygous subjects in each genotype group. Cell counts were normalised to 1.0 x 10^6^ cells at day 0 for all subjects due to variation observed in the number of cells obtained and to facilitate the day 6 comparison (S1 supplementary materials Table 1 in S1 File). None of the observed differences between the respective genotype comparisons were reported as statistically significant (i.e. a p-value > 0.05). Data is expressed as absolute cell count and shown as means ± SD.

Using the protocol optimised during our evaluation phase (see S1 supplementary materials pre-study protocol optimisation section in S1 File), isolated naïve CD4^+^ T cells (1.0 x 10^6^ cells for each condition) from each of the eighteen genotyped subjects (*i*.*e*. nine subjects from each homozygous genotype group), were cultured for six days under each of four growth medium formulations (S1 supplementary materials, Fig 1 in S1 File): T cell maintenance medium or Th17 driving medium in the presence of either GSK2794776A (an inactive diastereomer) or GSK2794778A (an inverse agonist of RORC- see methods section for further details). Comparison of total cell numbers on day 6 indicated no significant differences between the two genotypes for any condition (Fig 3). Likewise, differences between the four experimental conditions tested in the mean total cell counts for each genotype at day 6 were not statistically significant. These combined observations indicated that there were no statistically significant differences in the total T cell numbers that we could ascribe to genotype or any of the four experimental conditions. Relative proportions of cells expressing CCR6 were increased in all 4 conditions by TH17-driving medium compared to T cell maintenance medium (Fig 3), although the absolute proportion of CCR6+ CXCR3- cells was still low (less than 2.5% of total cells). This is comparable to published data using similar differentiation conditions [28] although methods of T cell differentiation using primed dendritic cells have yielded significantly higher numbers [29].

### Gene expression analysis

**Fig 4:**
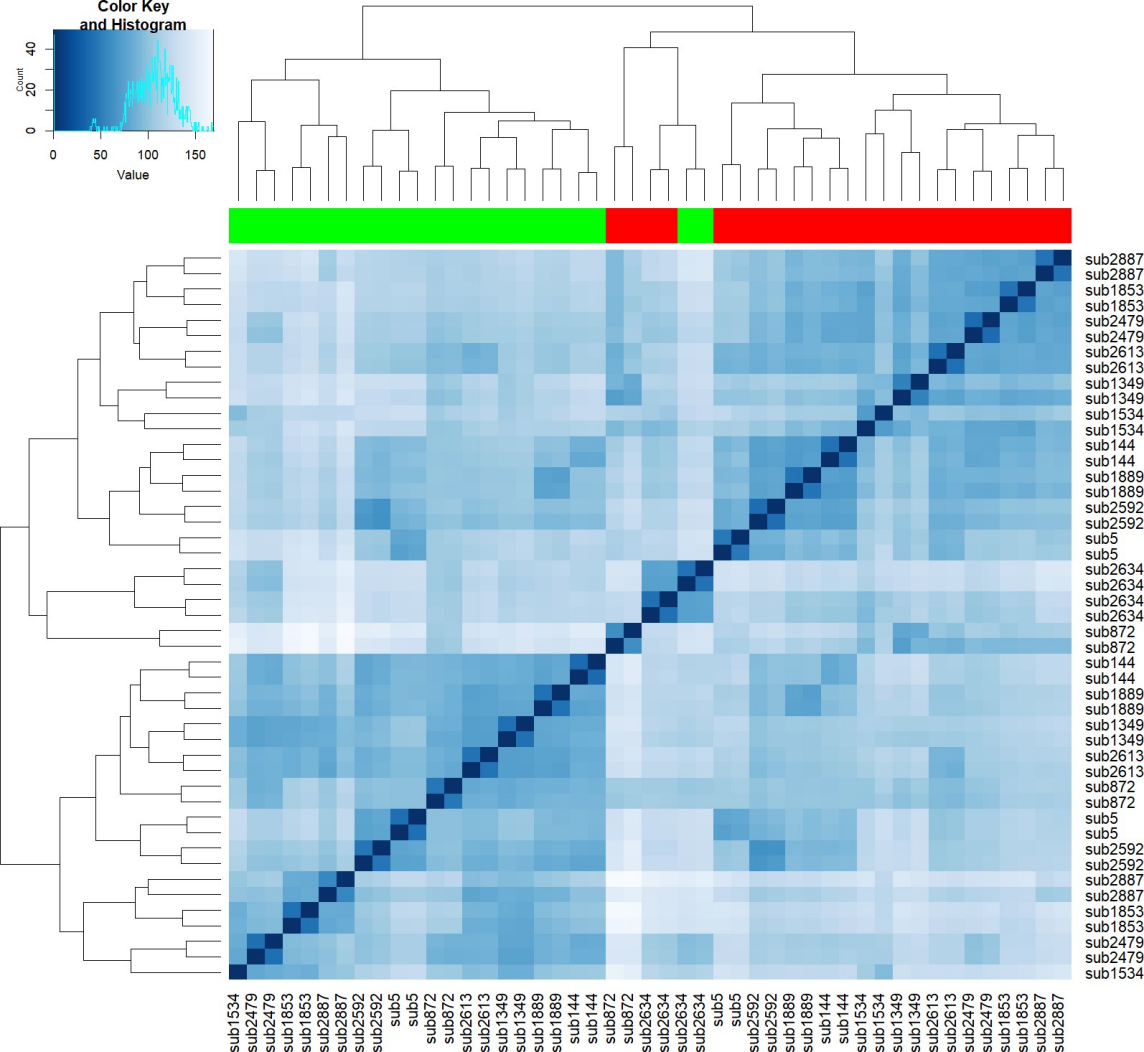
Transcriptional profiles cluster according to choice of differentiation medium. Distance matrix and clustering: The DESeq2 dist function was used estimate sample-to-sample Euclidean distances with regard to the normalised log-transformed gene expression profile, and a heatmap generated to visualise sample similarities. The dendrograms indicate that the profiles cluster primarily by differentiation medium (i.e. T cell maintenance cultured samples cluster together (indicated by the green bar), while Th17 stimulated samples group together (indicated by the red bar) and then by subject (i.e. within each incubation culture, samples from a subject are predominantly most similar to each other). Cell colour intensity correlates with the estimated distance between the two samples in question.

RT-PCR analysis of total RNA isolated from cell pellets collected at days 0 (baseline, before treatment), 1, 3, and 6 was used to quantify changes in expression of RORC and RORγt under the four experimental conditions (see the reverse transcriptase (RT)-PCR methods section and Fig 4 of S1 supplementary materials for further details in S1 File) and, in turn, to determine an optimum timepoint for whole genome RNA-seq analysis. Line plots of the normalised count values (S1 supplementary materials, Fig 4 in S1 File) indicated considerable changes in expression of both RORC and RORγt across the time-period under study. As previous studies had reported transcription details up to 3 days post RORC treatment [17,30,31] the longer incubation time was prioritised to both extend current public domain data and maximise downstream pathway analysis opportunities.

Whole genome RNA-seq data was generated from cells exposed to six days of each of the differentiation culture conditions using a standard workflow (see methods section for further details), and short reads mapped to the Ensembl human transcript GRCh37 database [32]. An average of 222x10^6^ counts were generated for each sample. Standard RNA-seq quality control (QC) metrics indicated that all samples except one (*i*.*e*. GG subject 6 –T cell maintenance medium with the inactive diastereomer) were broadly equivalent and of high quality (not shown). Exploratory high dimensional analysis of the log transformed counts indicated that samples clustered primarily by Th17 stimulus (Fig 4), with additional clustering in respect to subject and gender (not shown), and that these variables should be included in the subsequent statistical model (see methods) to correctly attribute variation in the observed data.

**Fig 5:**
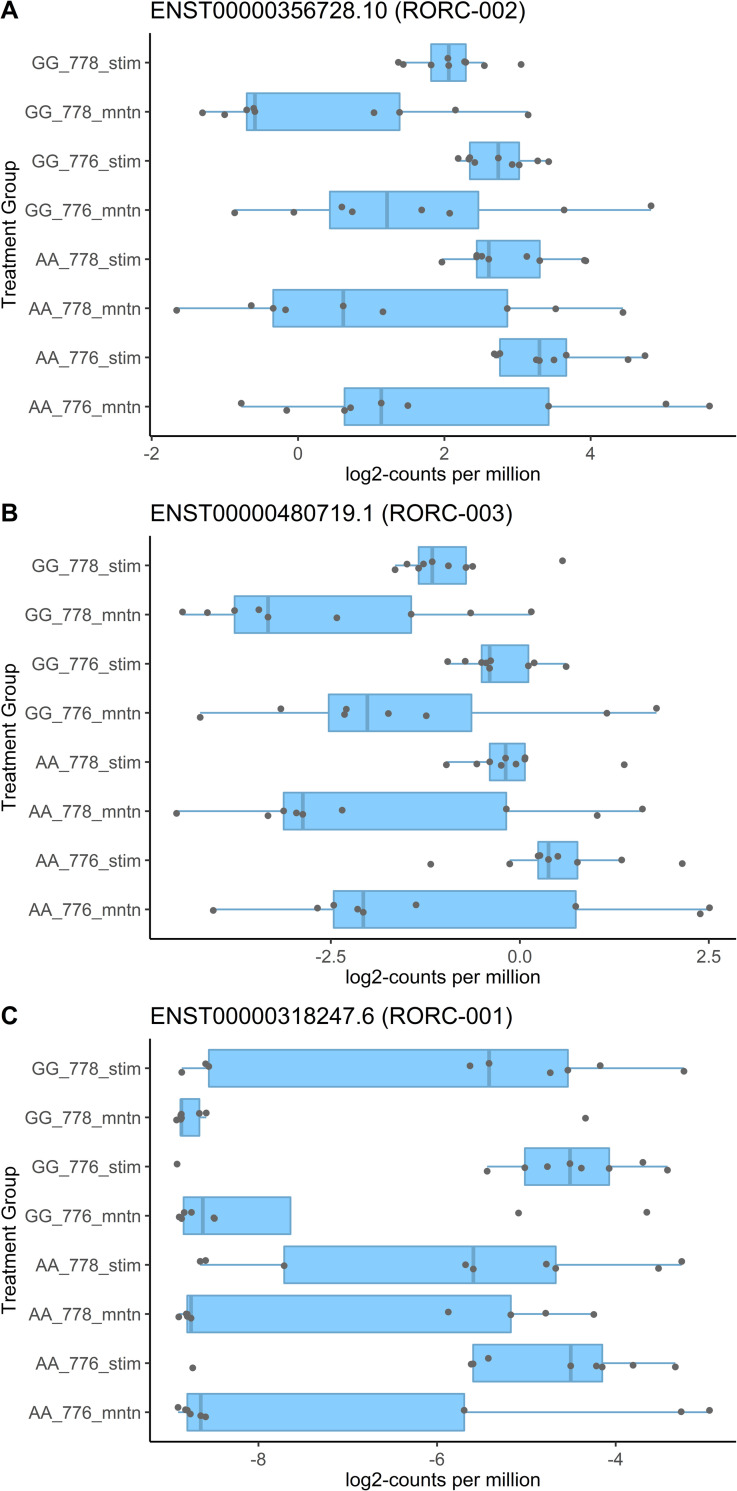
RORC transcripts are differentially expressed in presence of differentiating medium. Normalised of three RORC transcript counts. Each point represents the expression value observed in a subject and each box indicates the Q1, median, and Q3 quartiles for that differentiation medium group. AA & GG indicate the respective genotype of the subjects, 778 (the GSK2794778A inverse agonist) 776 (the GSK2794776A inactive diastereomer) indicate the presence of inactive and active molecules, while stim and mntn indicate TH17 and maintenance, respectively.

RNA-seq count data was used to compare expression levels of the four RORC transcripts reported in the Ensembl [32] transcript database. Comparative plots indicated that the RORγt (RORC-002) transcript is the most highly expressed under all conditions, and that this transcript is significantly upregulated following Th17 stimulation (Fig 5A). The non-coding (RORC-003) transcript was the next highest RORC transcript expressed and this transcript was also significantly upregulated following Th17 stimulation (Fig 5B). Expression of both transcripts was consistently lower in cells with rs4845604 GG relative to the AA genotype in the Th17 culture conditions, particularly in the presence of the inverse agonist. However, these visually apparent differences were not reported as statistically significant. The canonical RORC (RORC-001) transcript was expressed at very low levels (Fig 5C) while the nonsense mediated decay transcript (RORC-004) was not detected (not shown).

Expression of selected cytokine transcripts were evaluated for consistency (*i*.*e*. expression level and expression response to stimulus and agonist of TH17 signature genes) with previously reported Th17 differentiation studies such as Skepner (2014) and Castro (2017) [17,25]. For each transcript we observed a high degree of concordance with previous studies, for example, transcripts of IL17A-001, IL17F-001 CCL20 and IL23R-001 were each highly upregulated by Th17 stimulation and significantly reduced in the presence of the RORC inverse agonist (S1 supplementary materials, Fig 6 in S1 File). In contrast, expression of the JAK2-001 transcript was significantly reduced by Th17 stimulation but unaffected in the presence of the RORC inverse agonist (not shown).

### Differential expression and pathway analysis

Low expression transcripts were removed prior to statistical analysis, leaving 105,913 transcripts as input into the statistical model. A full-rank model was fitted and coefficients describing treatment, batch and sex used to obtain estimates of differential expression between subjects. Inter-correlation between samples originating from the same subject was controlled for using the limma [23] duplicate correlation function, which estimates this correlation by fitting a mixed linear model by Residual Maximum Likelihood Estimation for each transcript. Four statistical contrasts were made to prioritise significantly differentially expressed transcripts (see methods). Contrast_1 was considered the baseline comparison and was defined by contrasting samples of the GG and AA genotypes that were unstimulated and exposed to the inactive compound (GSK2794776A). Contrast_2 was defined by contrasting the two genotypes as for Contrast_1 in the presence of the RORC inverse agonist (GSK2794778A). Contrast_3 and Contrast_4 were defined using samples exposed to the Th17 stimulus in the presence of either the inactive compound or the RORC inverse agonist, respectively.

To better understand the likely power of the study, the expression values of selected Th17 signature genes [17,25] were used to quantify between- and within-subject variance in expression of the respective transcripts. Power estimates using medium-to-highly expressed transcripts, such as IL23A, indicated that the expression data had sufficient power to detect a 2-fold difference in expression (see methods section and S1 supplementary materials Fig 5A for further details in S1 File). In addition, Q-Q plots (S1 supplementary materials Fig 5B in S1 File) were used to assess the distribution of the p-values, reported with each of the differential expression results, and confirm that the smaller p-values deviate from what we would expect by chance alone. Following these two assessments, a joint threshold of >1 (absolute) log2 fold change, combined with an associated p-value of <0.01, was applied to the differential expression results to prioritise transcripts as input to enrichment analysis. The prioritised gene transcripts were then analysed using Qiagen’s Ingenuity Pathway Analysis platform [24] to identify enriched pathways and functions, and to predict putative upstream regulators of these pathways and functions. An average of 300 genes (detailed in S2_supplementary_DEG_lists section in S2 File) from each contrast were found to be eligible for pathway analysis.

**Fig 6:**
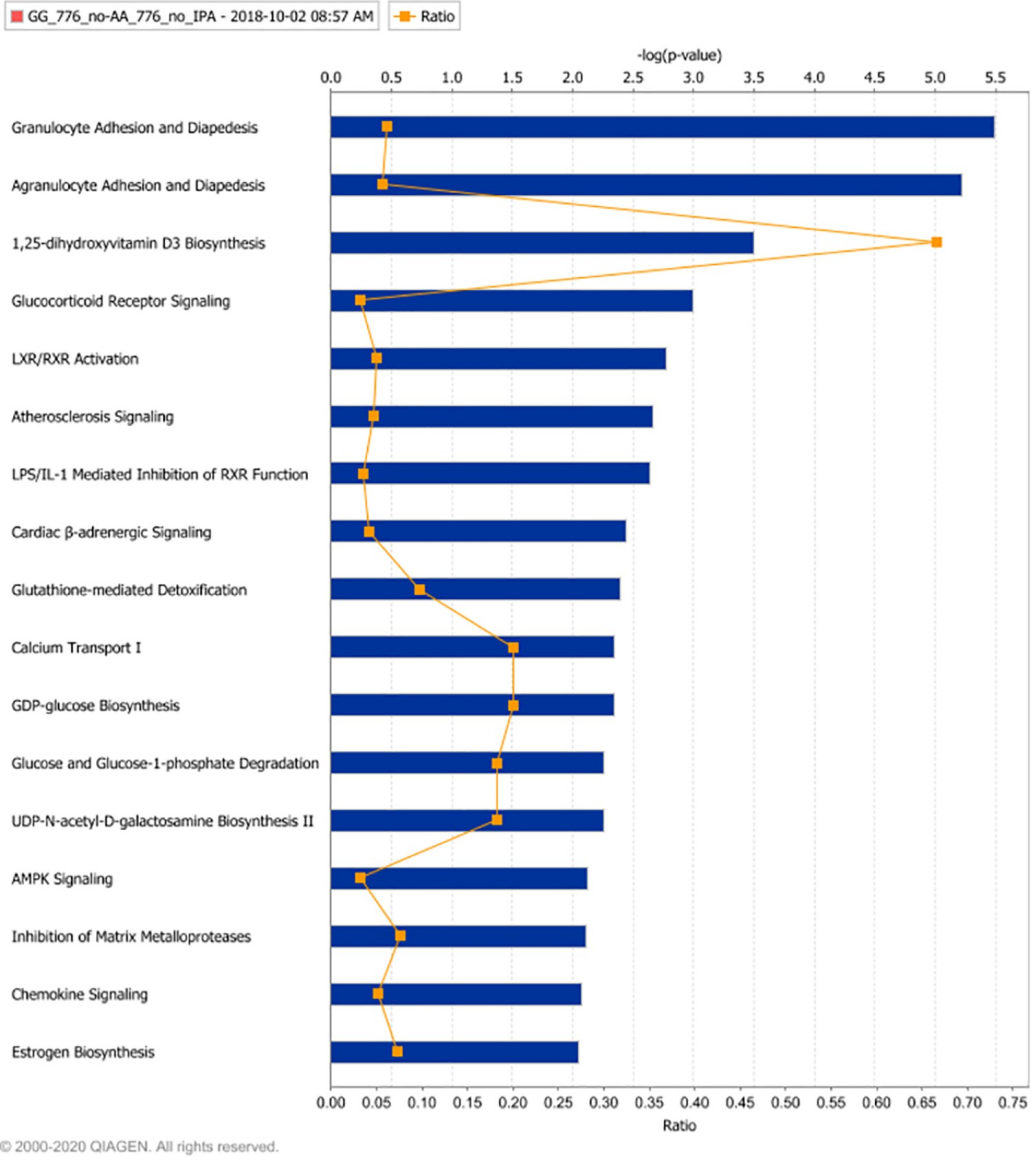
Genotype is associated with differential transcriptional effects on effects on canonical immune and metabolic signalling networks. Canonical Pathway enrichment analysis: Ingenuity pathway enrichment analysis was completed using those gene transcripts reported as significantly differentially expressed when cells isolated from GG genotype subjects cultured for six days in T cell maintenance medium in the presence of an inactive diastereomer (GSK2794776A) were contrasted with cells isolated form AA genotype subjects and subjected to identical culture conditions. Enriched pathways were identified and ranked using Fisher’s Exact Test. The blue bars represent the -log(p-value) of these tests (i.e. longer bars represent stronger evidence of enrichment). The ratio of the number of differentially expressed genes relative to the number of genes included in the pathway is indicated by the orange line.

Ingenuity’s Canonical Pathway analysis of the baseline differentially expressed profile (Contrast_1) identified Granulocyte & Agranulocyte Adhesion and Diapedesis as the most significant difference between subjects of the GG and AA genotypes (Fig 6). The differentially expressed genes associated with this prediction included CCL7(+), CCL8(+), CCL24(+), CCL25(+), CSF3R(+), IL1RN(+), ITGB2(+), MMP8(+), MMP12(+), SDC2(+) and TNFRSF1A(-): where a + or—symbol indicates up- or down-regulation in the GG genotype relative to the AA genotype, respectively. Other pathways reported as differentially affected between genotypes included: the LXR/RXR activation pathway, the 1,25-dihydroxyvitamin D3 Biosynthesis pathway and the Glutathione-mediated Detoxification pathway. The Ingenuity Functional Analysis tool reported differences in a Bacterial Infections function (based on differential expression of ADGRE5, C1QA, CAPG, CBY1, CCL8, CD1B, CHIT1, CLEC12A, DEFA1, F3, FCGR1A, GCA, IL1RN, IRF3, ITGB2, MMP12, MMP8, MSR1, MXI1, NSG1, POR, SERPINE1 and TNFRSF1A) as the most significant (associated p-value of 7.7E-08) functional difference between the two genotypes at baseline. Ingenuity’s Upstream Analysis tool predicted Fluticasone Propionate, a glucocorticoid anti-inflammatory agent, (p-value of 4.4E-10) and IL4 (p-value of 4.2E-08) as the most likely regulators of the observed differences in expression.

Comparing baseline expression in the presence of the RORC inverse agonist (Contrast_2) again identified Granulocyte & Agranulocyte Adhesion and Diapedesis as the most significant difference between subjects of the GG and AA genotypes (S1 supplementary materials Fig 7 in S1 File). The differentially expressed genes associated with this prediction included CCL7(+), CCL18(+), CCL25(+), FRP1(+), IL1RN(+), ITGB2(+), MMP8(+), MMP9(+), MMP12(+), MMP19(+) and TNFRSF1A(-). Other pathways reported as differentially represented included LXR/RXR Activation, Inhibition of Matrix Metalloproteases and LPS/IL-1 Mediated Inhibition of RXR Function. The Functional Analysis output reported differences in a Bacterial Infections function (associated p-value of 5.0E-15), Inflammatory response (9.1E-13) and Adhesion of blood cells (1.6E-10) as the most significant functional differences between the two genotypes at baseline. Ingenuity’s Upstream Analysis tool predicted that Fluticasone Propionate (p-value of 5.8E-09) and IL13 (p-value of 8.4E-09) as the most likely regulators of the observed differences in expression.

To better understand potential differential, genotype-driven transcriptional responses to Th17 stimulation the Ingenuity suite of analysis tools were applied to both Contrast_3 and Contrast_4. Canonical Pathway Analysis of the gene transcripts reported as differentially expressed by Contrast_3 (S1 supplementary materials Fig 8 in S1 File) reported a combination of inflammatory and metabolic signalling pathways, most notably, the LPS/IL1 mediated inhibition of RXR function (based on differential expression of FABP2 (+), FABP3 (+), GSTM1 (+), GSTM2 (+), GSTM3 (+), GSTT2/GSTT2B (+), IL1RAP (+), IL1RN (+), SCARB1 (+) and TNFRSF1A (-)) and the Glutathione-mediated detoxification pathways (see the glutathione associated genes highlighted above). Functional Enrichment analysis reported differences in signalling mechanisms related to Cancer (p-value of 1.0E-07), Apoptosis (p-value of 1.0E-06) and Tumorigenesis (p-value of 2.9E-06). Upstream Analysis predicted PAK1 (p-value f 2.4E-05), IL10RA (2.9E-05) and STAT3 (4.4E-05) as the most likely regulators of the observed differential expression profile.

**Fig 7:**
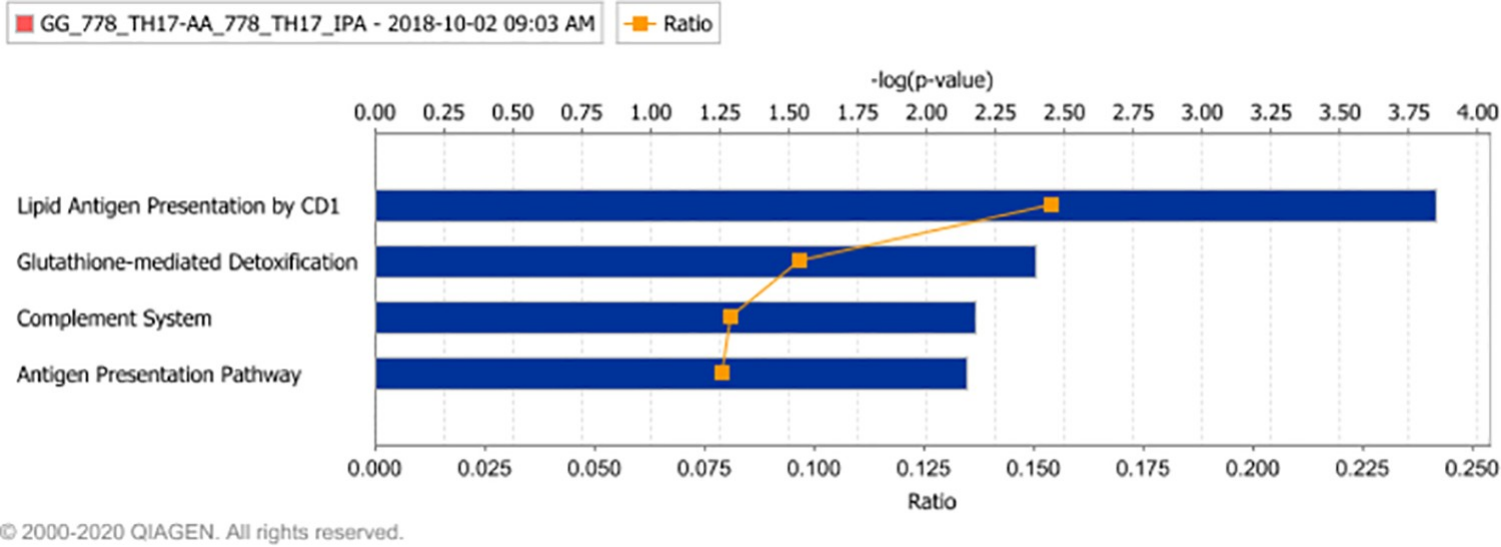
The homozygous genotype differentially effects transcriptional response in the presence of an RORC inverse agonist. Canonical Pathway enrichment analysis: Ingenuity pathway enrichment analysis was completed using those gene transcripts reported as significantly differentially expressed when cells isolated form GG genotype subjects cultured for six days in Th17 driving medium in the presence of an RORC inverse agonist (GSK2794778A) where contrasted with cells isolated form AA genotype subjects and subjected to identical culture conditions. Enriched pathways were identified and ranked using a Fischer’s Exact Test. The blue bars represent the -log(p-value) of these tests (i.e. larger bar size equates to statistical significance of the enrichment). The ratio of the number of differentially expressed genes relative to the number of genes included in the pathway is summarised by the orange line.

Canonical Pathway analysis for Contrast_4 reported differences in three immune signalling pathways (Fig 7): Lipid Antigen Presentation by CD1, (based on differential expression of AP2S1 (+), CD1A (+), CD1B (+) and CD1C (+)), the Complement System (given differential expression of C3 (+), CFH (-) and ITGB2 (+)), and the Antigen Presentation Pathway (given differential expression of CIITA (-), HLA-G (+) and NLRC5 (-)). The Glutathione-mediated Detoxification pathway was again reported as likely to be perturbed (given differential expression of GSTM1 (+), GSTM3 (+) and GSTO1 (-)). Functional Analysis identified several genotype differences relating predominantly to Cancer signalling (1.1E-06:1.1E-04) and Antigen presentation of Lipid (supported by the CD genes listed above). The Upstream analysis tool predicted that the cytokine IL4 (p-value 1.6E-04) and transcriptional regulator TCL1A (p-value 3.7E-04) were the most likely regulators of the observed differential expression profile.

Twenty-two transcripts were reported as significantly differentially expressed in all four contrasts (S1 supplementary materials Table 2 in S1 File) and only one Canonical Pathway, the Glutathione-mediated Detoxification pathway, was consistently reported. Ingenuity’s Upstream Analysis tool predicted IL4 (1.7E-04), IL10RA (5.1E-04) and Fluticasone Propionate (5.3E-04) as potential regulators of the reported differential expression profile in all four statistical contrasts.

To determine the robustness of the reported pathway analysis we removed all transcript identifiers from the count table and then randomly assigned these labels to the unmodified count data table (i.e. complete disassociation of all labels while retaining integrity of the count table) 10 times and completed pathway analysis with each of these permuted data sets. In all 10 permuted pathway analyses the p-value of the respective most significant pathways reported (for the four contrasts discussed) was at least one order of magnitude greater than the p-value observed with the original unpermuted data. For example, pathway analysis of contrast 1’s differentially expressed transcript list predicts the Granulocyte adhesion & Diapedesis pathway as the most significant pathway, with an associated p-value of 2.07E-06. After permuting the dataset (10 times) and repeating the pathway analysis the most significant pathway reported (of the 10 permutations) was the Synaptogenesis Signalling Pathway with an associated p-value of 2.96E-04. This indicates that the pathway analysis has captured a transcriptional effect that is more significant than would be expected by chance.

## Discussion

The Th17 cell lineage plays a significant role in autoimmune and inflammatory conditions such as arthritis, asthma and inflammatory bowel disease [33–35]. Given that the RORγt nuclear receptor directs both the differentiation of naïve CD4+ T and the expression of pro-inflammatory cytokines [36,37] it is considered an important therapeutic target in the treatment of such conditions [8–10]. Independent GWAS and PheWAS studies reported significant associations between the RORC regulatory variant, rs4845604, and a protective effect for several allergic traits [14,16]. To better understand this finding, and the possible dependence of any differences observed on activation of pathways that drive naïve T cells towards TH17 differentiation, and specifically on activity of RORγt itself, we investigated both the cellular differentiation and the transcriptional response of purified naïve CD4+ T cells from healthy subjects homozygous for either the reference (G) or minor (A) allele of rs4845604 under four controlled experimental conditions. Thus, isolated naïve T cells were differentiated in the presence and absence of a cytokine cocktail optimised to drive TH17 production. Both the maintenance and TH17 differentiation conditions were tested in the presence of the RORC inverse agonist GSK2794778A (to prevent constitutive receptor activity), or its inactive diastereomer GSK2794776A (as a control). We set out to determine whether rs4845604 genotype (1) affected T-cell numbers, (2) was associated with transcriptional signature at baseline or after TH17 stimulation, and (3) if transcriptional effects are observed, predict what downstream biological pathways are likely to be affected.

Baseline total cell counts indicated that rs4845604 genotype had no effect on naïve CD4+ T cell numbers in healthy subjects. This is a relatively small pilot study and it is possible that variant driven changes would only emerge with larger subject numbers: however, following 6 days incubation, no statistical difference in total cell number could be attributed to genotype or active compound in any of the four culture conditions. These findings indicate that the inverse agonist is non-toxic in this setting, an observation that concurs with previous reports [15,17,25,30,31,38], and that the RORC variant has no significant effect on CD4^+^ T cell viability.

Further cell count analysis, focussing specifically on the Th1-like and Th17-like cell populations, indicated that 6 days incubation in maintenance medium resulted in a significant increase in the percentage of Th1-like cells, while the presence of the RORC inverse agonist significantly reduced the percentage of Th17-like cells retrieved: again, a response that has previously been reported [2,25,31,38,39]. However, statistical analysis of the Th1-like and Th17-like cell count data indicated that the rs4845604 variant has no significant effect on the number of either cell type detected, under any of the four culture conditions.

Given that our primary interest was in the RORγt transcript and that the rs4845604 variant mapped to an upstream, non-protein coding region, we considered it most likely that any genotype effect would manifest as transcriptional changes. Exploratory analysis of RNA-seq data unambiguously clustered the samples by culture condition and subject, giving assurance that the expression data had robustly captured features of the experimental design. Furthermore, comparative analysis of the normalised transcript count data for Th17 signature genes, confirmed previously reported biological characteristics. For example, IL17A, IL17F, CCL20 and IL23R were all observed to be significantly up-regulated following stimulation, while JAK2 and TYK2 were clearly down-regulated following Th17 stimulation, and both IL17A and IL17F were significantly down-regulated in the presence of the RORC inverse agonist. The magnitude of the observed transcriptional dysregulation was in broad agreement with previous reports [17,30] and adds further confidence to current understanding of the Th17 differentiation process. These data are confirmatory that significant differentiation towards a TH17-like phenotype is induced by TH17 driving medium, despite the low absolute numbers of CCR6+ cells detected following incubation.

The Ensembl database indicates that the RORC gene can be expressed as four unique transcripts. Our expression data provides evidence that three of the four transcripts are expressed in either, or both, naïve CD4+ T cells or Th17 stimulated cells, and that both the RORγt (RORC-002) and a non-coding transcript (RORC-003) are most highly expressed under all four culture conditions while the canonical (RORC) transcript was close to undetectable in naïve cells. RORC canonical, RORγt and RORC non-coding transcripts were clearly up-regulated under Th17 stimulating conditions, and as the RORγt transcript was expressed at 2000-fold greater levels than the canonical transcript, this emphasises that importance of RORγt in Th17 cell differentiation.

Combined, the robust, coherent clustering of the sample data and the close agreement with previous RORγt treatment studies supported our conclusion that the expression data was of sufficient quality to facilitate pathway analysis. A robust statistical model was used to detect changes in expression that could be attributed to the variant under each of the four culture conditions. This analysis indicated that none of the four RORC transcripts were reported as significantly differentially expressed in any of the applied statistical contrasts, indicating that rs4845604 genotype had no significant differential effect on RORC transcription under the four evaluated culture conditions.

Treatment with the RORC inverse agonist resulted in transcriptional changes that concur with previous studies [17,37] and support our understanding that the compound is exerting its transcriptional effects via structural changes in RORC protein conformation that favour recruitment of co-repressors and negating association of co-activators. Rather than focus on (potentially spurious) individual gene changes, we applied a combination of enrichment analysis tools to better understand the most likely effect of rs4845604 genotype on known pathways. Pathway analysis of the baseline (*i*.*e*. no stimulus and no inverse agonist) differential expression profile identified significant differences in both immune and metabolic pathways that could be attributed to rs4845604 genotype. Perturbation of “granulocyte adhesion and diapedesis”, and “vitamin D3 biosynthesis” were the top pathways reported. The former prediction is an interesting observation as IL17A has been reported to promote granulocyte recruitment [40], infiltration [41] and food allergy conditions [42]. However, none of the IL17 family members were reported as differentially expressed and the granulocyte pathway perturbation was attributed to differential expression of several genes, that included; TNFRSF1A, IL1RN, MMP8, MMP12, CCL25, CCL24 and CCL7. The possibility that vitamin D biosynthesis may be perturbed is also an intriguing observation as vitamin D is reported to down-regulate both pathogenic Th17 cell markers and IL17A secretion in healthy human donors [43] and suppress stimulated CD4+ T cell proliferation capacity [44]. To better understand how these and other pathway perturbations may have originated, we used the Ingenuity upstream regulator analysis tool to analyse the baseline differential expression profile. This analysis identified strong similarities with the expected effects on known downstream targets of both fluticasone propionate and IL4. The latter is particularly interesting as neutralisation of IL4 has been shown to induce Th17 inflammation in a preclinical asthma model [45], while a variant of the IL4RA gene was reported to favourably induce Treg cells to the Th17 cell fate [46]. Details of how rs4845604 could be perturbing any of these mechanisms will require further investigation, however, it should be noted that the data underlying this analysis was derived from a small sample and that the inferred associations should be treated with a degree of caution, but they do offer biologically plausible starting points for future studies.

Pathway analysis of the differential expression profile at baseline in the presence of the RORC inverse agonist also identified immune and metabolic pathways—several of which were reported in the baseline contrast. This indicates that under baseline conditions, the inverse agonist invokes few effects on transcription that can be attributed to the respective homozygous genotypes, as most of the pathways reported as perturbed in the presence of the inverse agonist are also reported as perturbed in the presence of the inactive compound. An interesting exception is the absence of the 1,25 dihydroxy vitamin D3 biosynthesis pathway in the presence of the RORC inverse agonist, as perturbation of this pathway was one of the top hits in the baseline analysis. There are no reports detailing direct regulatory interactions between CYP27B1 (the gene that encodes 25-hydroxyvitamin D3-1-alpha-hydroxylase (1-alpha-(OH)ase) and reported as differentially expressed in this study) and RORC, but activated T cells are known to express CYP27B1 and synthesise sufficient vitamin D to affect vitamin D-responsive genes [47]. Our data suggest that the rs4845604 variant may affect T cell vitamin D biosynthesis and possibly T cell differentiation fates, and that treatment with the RORC inverse agonist ameliorates this effect.

A further statistical contrast (contrast_3) was used to identify rs4845604-directed changes following Th17 stimulation in the presence of the inactive diastereomer. Pathway enrichment analyses of this differential expression profile predicted perturbation of LPS/IL1-mediated inhibition of RXR as the most significant rs4845604 genotype-associated difference. This is an interesting observation as this pathway details the RXR (retinoid X receptor) regulatory effects on the innate immune response to gram negative bacteria endotoxins, and RORγt is a critical regulator of anti-microbial immunity [3,8]. If validated, this rs4845604-associated signalling event could have a significant impact in the elucidation of novel disease risk factors.

Pathway analysis of the differential expression profile following culture in the presence of the Th17 stimulus and RORC inverse agonist identified the fewest canonical pathways, suggesting that the observed rs4845604-associated effects were considerably reduced in the presence of the RORC inverse agonist: The top hit associated with this differential expression profile was the lipid antigen presentation by CD1 pathway. The CD1 family, though poorly understood, comprise a conserved family of lipid-presenting antigens implicated in both infectious and autoimmune diseases [48]. Perhaps of relevance to this study is that the CD1A inflammatory response is mediated by Th17 cells [49] and that in vivo anti-IL17A treatment has been observed to ameliorate skin inflammation in a mouse psoriasis model [50]. Our data suggest that rs4845604 genotype affects CD1 pathway signalling, which could possibly equate to a clinically significant effect under certain physiological conditions.

In all four statistical contrasts the Glutathione-mediated detoxification pathways were reported, which suggests a pronounced rs4845604 genotype effect irrespective of other cellular responses. The glutathione S-transferases (GSTs) comprise a family of detoxification enzymes that protect cells from intracellular reactive oxygen species [51], and GST genetic variability has been associated with susceptibility to a variety of toxins and an increase in certain cancers [52,53]. How the RORγt rs4845604 variant could regulate these enzymes is currently unknown, but the ROR family have been observed to regulate several enzymes involved in the metabolism of both synthetic and endogenous xenobiotic molecules [1], so a variant associated detoxification effect is biologically plausible. Given that all the differentially expressed GST enzymes observed in this study were significantly up-regulated in GG homozygous subjects, relative to the AA homozygous subjects, suggests that the GG genotype may elicit an increased cellular oxidative stress response: and that the GST enzymes are up-regulated to protect the cells from oxidative damage.

In summary, we have completed a proof of concept study to evaluate how the RORC rs4845604 variant may affect *ex vivo* CD4^+^ T cell differentiation and the transcriptional response of purified naïve CD4+ T cells under four controlled experimental conditions. Our analysis identified potentially novel functional effects of a disease-associated variant in the RORC gene that could plausibly contribute to perturbation of several immune and metabolic signalling networks. Our study justifies further investigation of the mechanism underlying the effects we have observed, in larger cohorts of both healthy subjects and individuals with autoimmune disease.

## Conclusions

In this proof of concept study we have investigated both the cellular differentiation and the transcriptional effects of the RORC variant rs4845604 in purified naïve CD4^+^ T cells under maintenance and Th17 stimulation culture conditions both in the presence and absence of an RORC inverse agonist. Pathway enrichment analysis of the differential transcript expression profiles identified novel, biologically plausible effects on immune and metabolic signalling networks. The observed data indicate that the homozygous rs4845604 GG genotype, associated with IBD risk may be associated with an increased detoxification response mediated by increased expression of multiple glutathione S-transferases.

## Supporting information

S1 File Supplementary experimental methods. (PDF)

S2 File The complete statistical summary statistics of the differential expression analysis. (XLSX)
