# Supplementary material for: Transcriptomic effects of rs4845604, an IBD and allergy-associated RORC variant, in stimulated ex vivo CD4+ T cells
Source: PLoS One. 2021 Oct 21;16(10):e0258316. doi: 10.1371/journal.pone.0258316 (PMC8530322; doi:10.1371/journal.pone.0258316)
Supplement: S1 File — (PDF) [file pone.0258316.s001.pdf]

Supplementary Materials

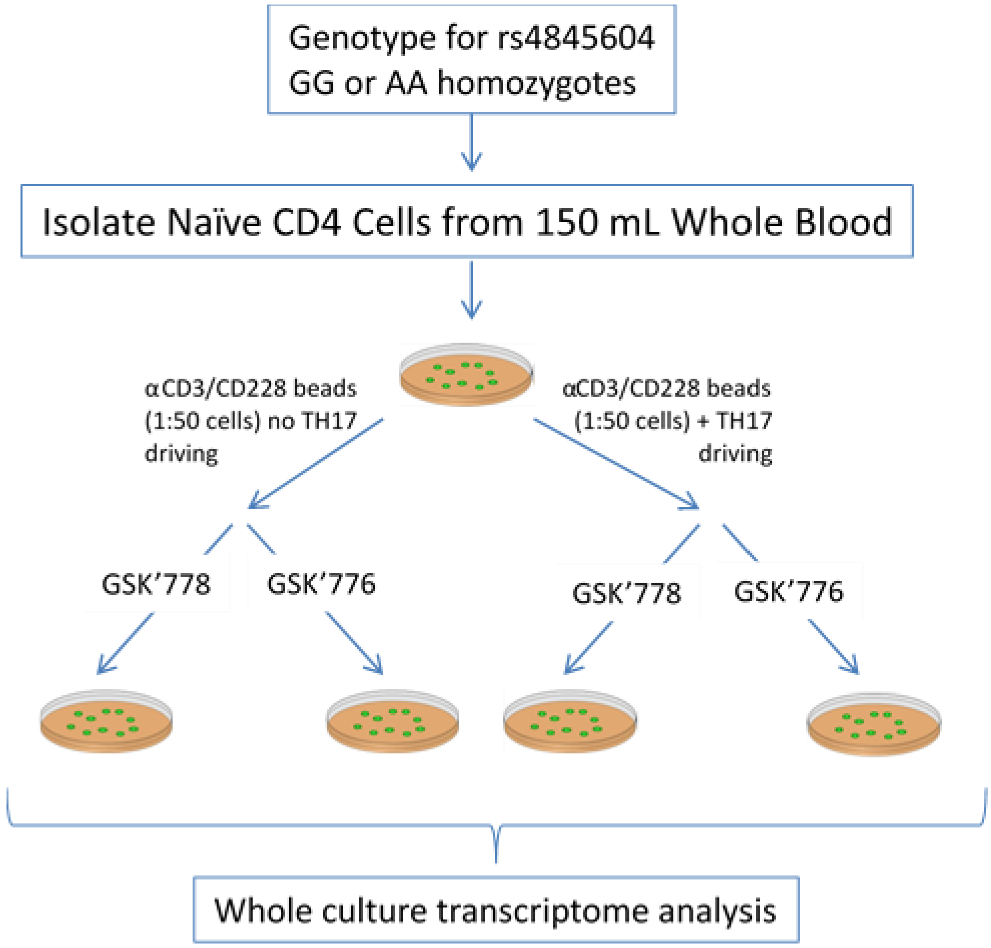

***Figure 1**: Study design schematic: purified naïve CD4 cells were isolated from rs4845604 GG or AA homozygote subjects and cell differentiation induced by incubating in one of 4 culture conditions (+/- TH17 driving medium, in the presence of either GSK'776 (GSK2794776A an inactive diastereomer) or GSK'778 (GSK2794778A an RORgt inverse agonist) for 6 days before commencement of transcriptome analysis.*

Pre-study protocol optimisation

To better understand how naïve CD4+ T cells respond to prolonged incubation in T cell maintenance and Th17 driven mediums, blood drawn from two healthy subjects (not included in the genotyped experimental studies described in the following sections) was used to complete an evaluation phase that would direct subsequent analysis of the genotyped T cells. Naïve $CD4^+$ T cells were purified from PBMCs with an estimated purity of 93.75 ± 3.5 % (*i.e.* the percentage of $CD45RA^+$ cells in the $CD4^+CD3^+$ population) when assessed by flow cytometry (supplementary figure 1). Note that naïve $CD4^+$ T cells were selected in preference to either the PBMCs or $CD4^+$ T cell elutes, despite there being a higher percentage of Th17 cells (at day 0) in both these cell populations (*i.e.* 0.25 % of isolated naïve $CD4^+$ T cells were Th17 [$CD3^+CD4^+CXCR3^-CCR6^+$], in comparison to 11.5 and 12.0 % isolated from PBMC and $CD4^+$ T cell elutes, respectively), as a primary interest of our investigation was to elucidate genotype specific effects of GSK2794778A (an RORC inverse agonist) on the differentiation of naïve $CD4^+$ T cells to Th17.

A preliminary flow cytometry investigation detailed how naïve $CD4^+$ T cells respond to six days of incubation in either T cell maintenance or Th17 driven mediums (*i.e.* a medium that stimulates *in vitro* polarisation of naïve CD+ T cells toward the Th17 lineage). Briefly, we observed (supplementary figure 2) that the percentage of naïve $CD4^+$ T cells ($CD4^+CD3^+CD45RA^+$) decreased under both medium conditions (from 98.6 ± 1.7 % at day 0, to 73.1 ± 10.2 % in Th17 driving medium, and to 29.4 ± 18.8 % in T cell maintenance medium, at day 6). The numerical difference between the observed decreases was not reported as statistically significant (*i.e.* a reported p-value > 0.05). In the same samples the Th17 population [$CD4^+CXCR3^-CCR6^+$] was observed to increase after 6 days of culture in the Th17 driven medium (from 0.46 ± 0.12 % to 4.44 ± 2.17 %). No major changes in Th17 number occurred following 6 days incubation in the T-cell maintenance medium (*i.e.* a difference of 0.13 ± 0.08 % was observed). The difference between these day 6 values was not reported as statistically significant. A more pronounced difference was observed in the Th1 population [$CD4^+CXCR3^+CCR6^-$] (from 2.99 ± 4.2 % to 69.1 ± 7.5 %) following 6 days of incubation in T cell

maintenance medium. While under Th17 driven conditions the increase in the Th1 population was considerably less pronounced (*i.e.* 17.4 ± 4.9 %). The difference in Th1 numbers under the different incubation conditions was reported to be statistically significant (*i.e.* a p-value < 0.05).

These combined observations indicate that incubation in Th17 driven medium marginally stimulates differentiation of naïve CD4$^+$ T cells to Th17, while significantly reducing differentiation to Th1 cells. The equivalent incubation in T cell maintenance medium favoured differentiation of naïve CD4$^+$ T cells to Th1 cells.

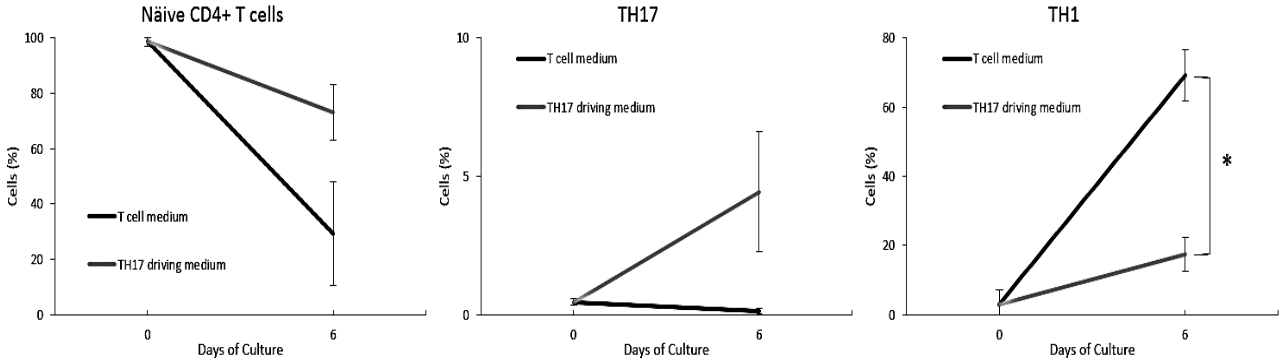

Figure 2: *Preliminary flow cytometry analysis of naïve CD$^+$4 cells isolated from (un-genotyped) healthy subjects (n=2), not included in the genotyped subjects experimental studies, indicated that incubation in Th17 driven medium lead to a decrease in the number of the naïve CD4$^+$ T cells: although this reduction was less than that observed following incubation T cell maintenance medium (left). A slight increase in Th17 cell number was observed following incubation in the Th17 driving medium, though the difference between medium conditions was not reported as statistically significant (middle). Th1 cell populations increased following incubation in both mediums and the difference between the respective increases was reported as statistically significant (right). Note that an asterix indicates a statistically significant (p-value < 0.05) difference.*

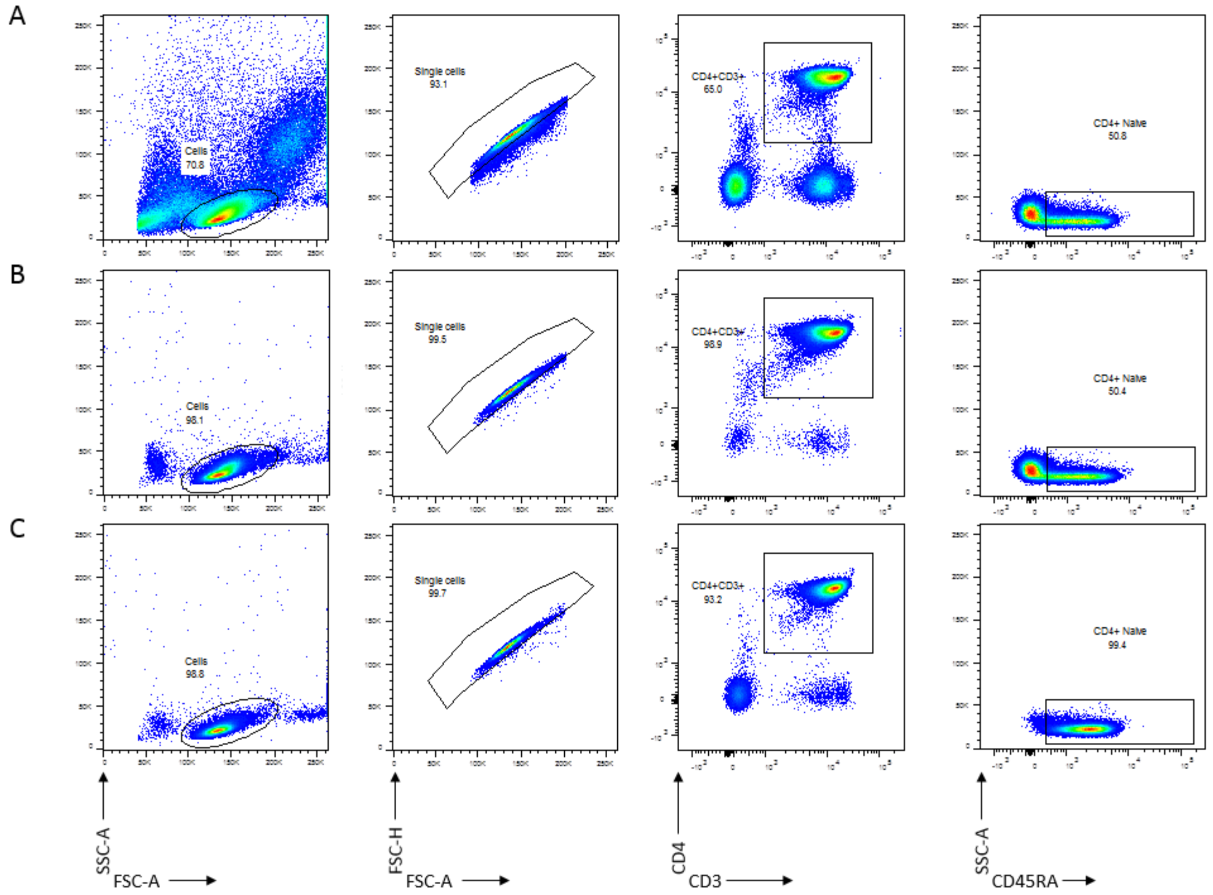

Figure 3: *Gating strategy used for the selection of naïve CD4*$^+$ *T cells (i.e. CD4*$^+$*CD3*$^+$*CD45RA*$^+$*) of (A) PBMCs, (B) CD4*$^+$ *cells, and (C) naïve CD4*$^+$ *T cells at day 0 of culture. As a primary purpose of our study was to analyse the effect of GSK279478A (an RORC inverse agonist) on the differentiation of naïve CD4*$^+$ *T cells to Th17, naïve CD4*$^+$ *T cells were selected in preference to PBMCs and CD4*$^+$ *T cells, despite the lower number of Th17 cells. Naïve CD4*$^+$ *T cells were isolated from PBMC cells with an estimated purity of 93.75 ± 3.5 %. Note that this preliminary flow cytometry was completed using blood isolated from healthy subjects and not from the genotyped subjects.*

Genotype	Subject ID	Ratio Naïve CD4+ T cells / PBMCs (%)	Mean ± SD
	2	5.06	
	5	13.75	
	320	1.91	
	886	11.54	
**AA**	1349	4.47	7.63 ± 4.07
	1534	4.48	
	1889	11.70	
	2479	9.24	
	2613	6.56	
	144	10.22	
	217	5.78	
	872	3.75	
	1287	12.22	
**GG**	1476	6.67	8.15 ± 3.83
	1853	5.15	
	2592	13.76	
	2634	11.69	
	2887	4.10	

Table 1: *Naïve CD4+ T cells were isolated from PBMCs from a total of 18 subjects, 9 from each genotype group (i.e. homozygous for AA or homozygous for GG). The difference in numbers of naïve CD4+ T cells (normalised relative to the number of PBMCs) was not statistically significant (p-value > 0.05). Note that only one count could be completed for each subject.*

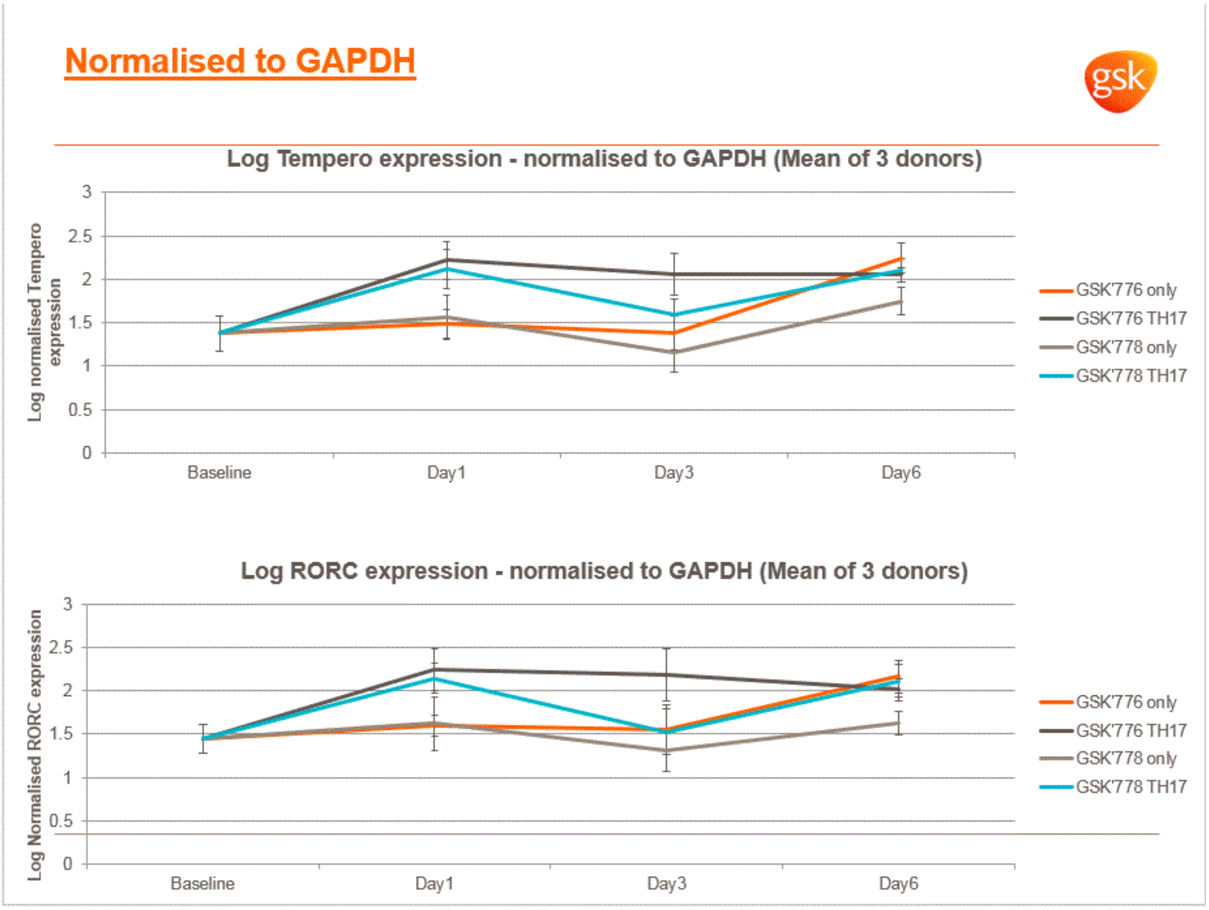

Figure 4: *RT-PCR analysis of total RNA isolated from cell pellets collected at days 0 (baseline), 1, 3, and 6 was used to quantify changes in expression of RORC (lower plot) and RORγt (upper plot) under the four experimental conditions: see the reverse transcriptase (RT)-PCR methods section in the main manuscript for further details of the primers used and the experimental conditions used. The resulting quantification values were used to determine an optimum timepoint for whole genome RNA-seq analysis. The legends refer to the small molecules GSK'776 (GSK2794776A an inactive diastereomer) or GSK'778 (GSK2794778A an RORgt inverse agonist)). TH17 indicates cells cultured in the Th17 driving medium (i.e. a medium that stimulates in vitro polarisation of naïve CD+ T cells toward the Th17 lineage).*

Fit Group

Response TH1 (%)

Whole Model

Effect Summary

Source	LogWorth	PValue
medium	4.317	0.00005
medium*Genotype	0.320	0.47849
medium*compound*Genotype	0.271	0.53605
compound	0.209	0.61858
compound*Genotype	0.171	0.67497
medium*compound	0.080	0.83230
Genotype	0.079	0.83417

Fit Group

Response TH17 (%)

Whole Model

Effect Summary

Source	LogWorth	PValue
compound	1.464	0.03434
Genotype	1.065	0.08613
medium	1.028	0.09376
medium*compound*Genotype	0.514	0.30625
medium*compound	0.306	0.49411
medium*Genotype	0.137	0.72936
compound*Genotype	0.001	0.99759

Figure 5A: *To assess differentiation responses of naïve CD4+ T cells (in the respective differentiation mediums) and determine the most suitable timepoints for sampling in the main expression study, cells from six genotyped subjects (3 subjects from each genotype group) were analysed using flow cytometry. The difference in subsequent cell counts were evaluated by ANOVA (i.e. Fixed Effect Tests). The ANOVA for Th1-like (%) reported a highly significant effect on Th1-like numbers dependent on the medium used, but no significant effect for any other factor, or the interactions between the factors. The LogWorth axis represents the -log10(p-value) and a longer bar indicates a model term with a more statistically significant effect. The blue vertical reference line corresponds to p = 0.01*

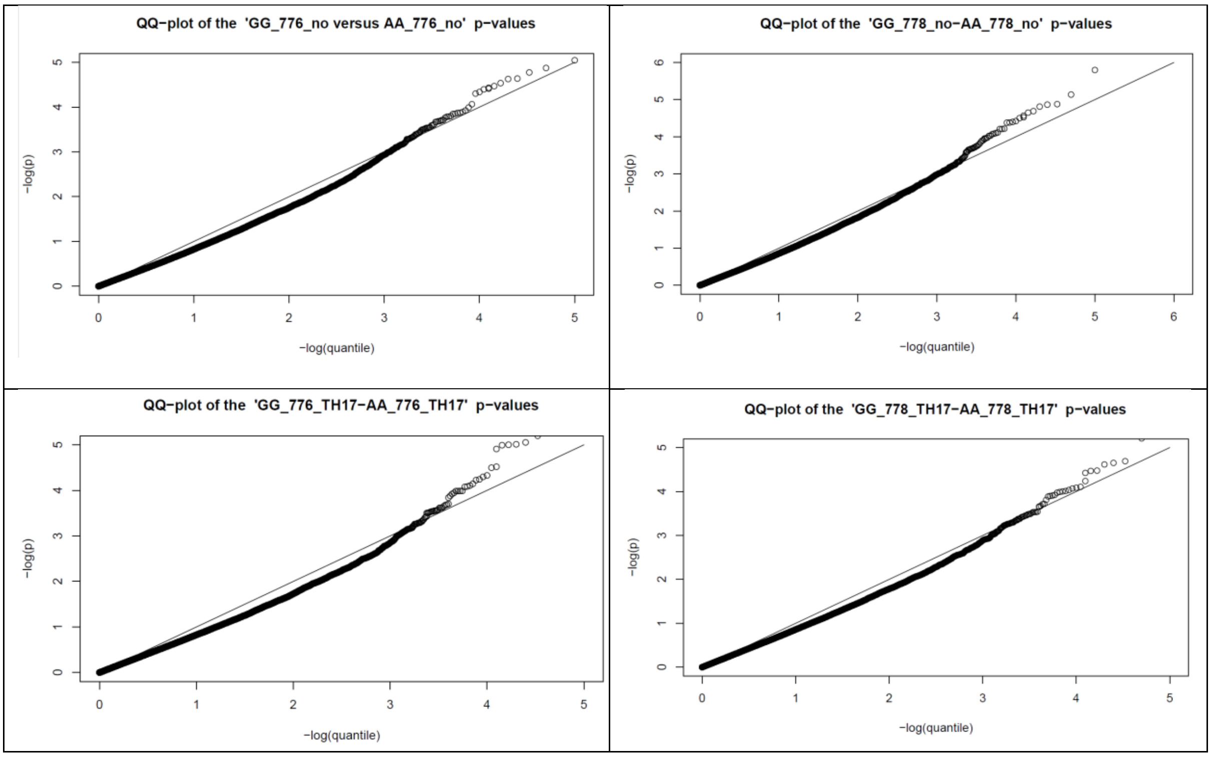

Figure 5B: *Q-Q plots of the p-values reported with the four statistical contrasts (see methods section in the main manuscript for further details of the statistical model used). In each plot there is a clear deviation from the theoretical quantile, supporting our assertion that the smallest p-values deviate from what we would expect to see by chance alone, and that the associated transcripts are suitable for input to pathway analysis. Subject genotypes are indicted as GG or AA. While 776 and 778 indicate GSK2794776A (an inactive diastereomer) or GSK2794778A (an RORgt inverse agonist). Samples cultured in T cell maintenance medium are labelled no, while samples cultured in Th17 driving medium are labelled TH17.*

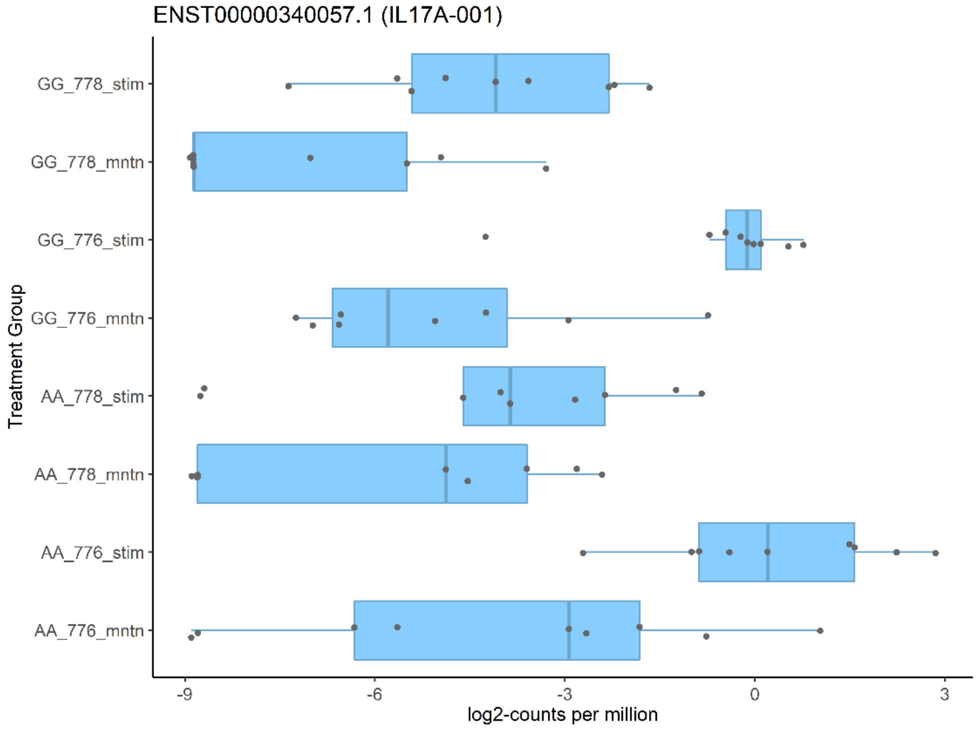

ENST00000340057.1 (IL17A-001)
GG_778_stim
GG_778_mntn
GG_776_stim
GG_776_mntn
AA_778_stim
AA_778_mntn
AA_776_stim
AA_776_mntn
Treatment Group
-9
-6
-3
0
3
log2-counts per million

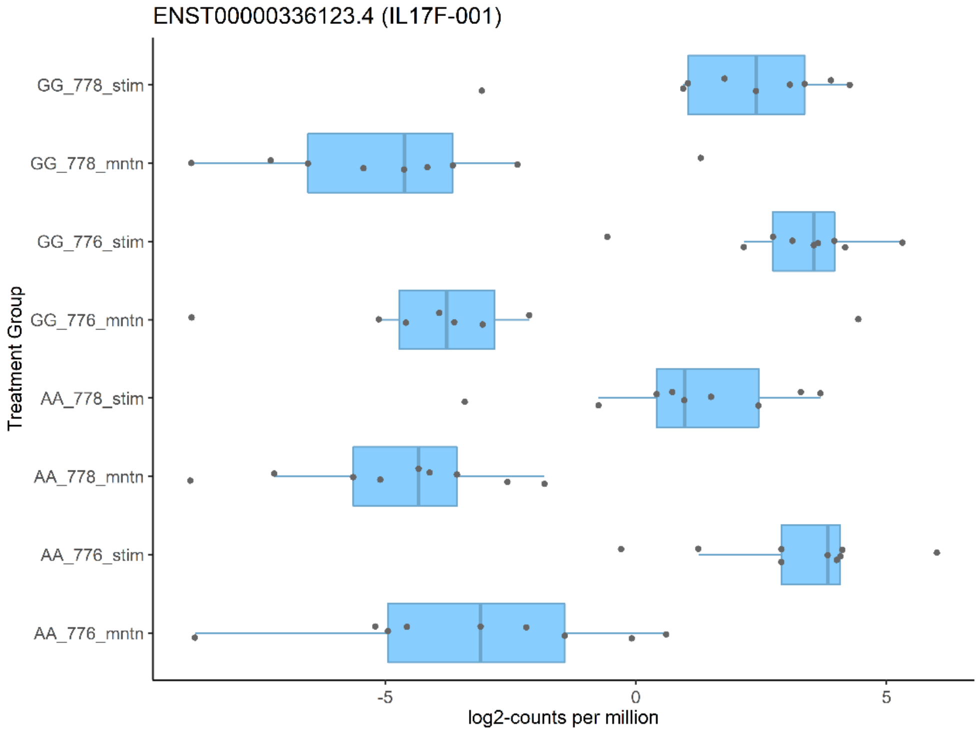

ENST00000336123.4 (IL17F-001)
GG_778_stim
GG_778_mntn
GG_776_stim
GG_776_mntn
AA_778_stim
AA_778_mntn
AA_776_stim
AA_776_mntn
Treatment Group
-5
0
5
log2-counts per million

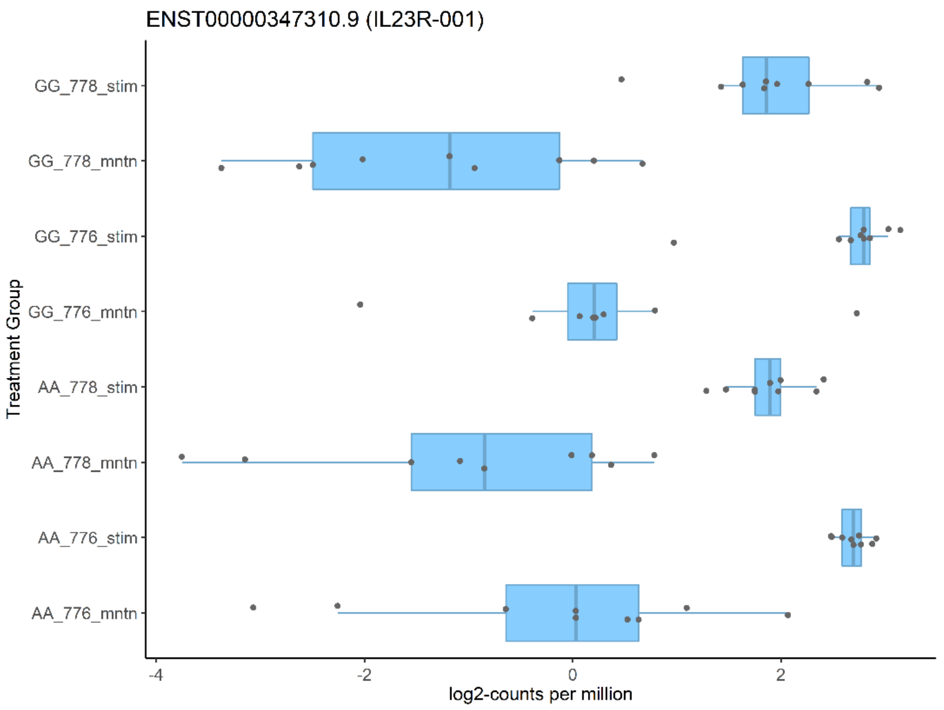

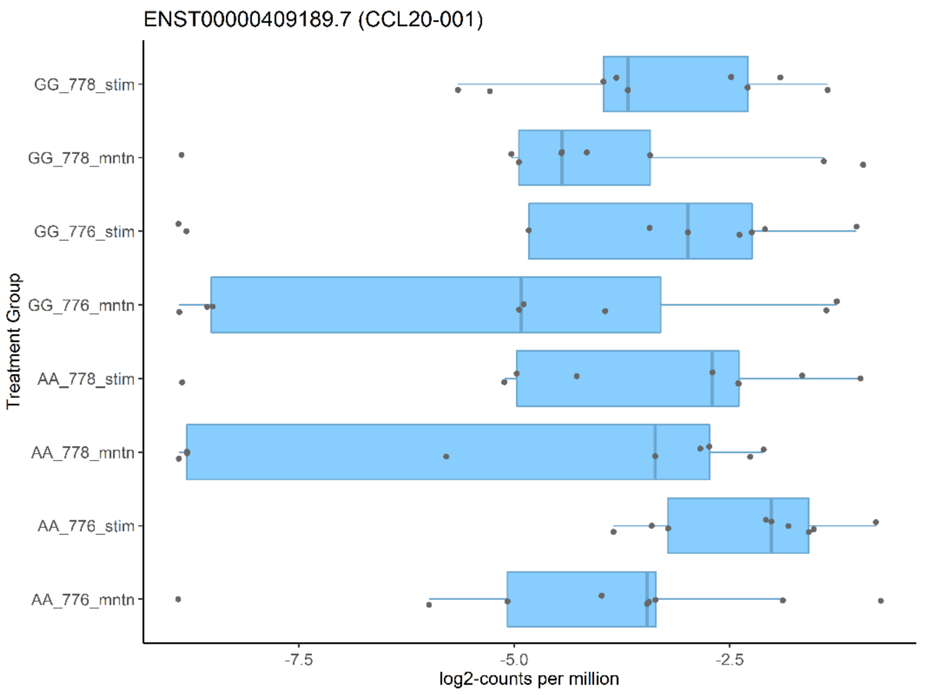

Figure 6: *Normalised transcript counts of cytokines previously reported to be differentially expressed during Th17 maturation. Each point represents the expression value observed in a subject and each box indicates the Q1, median, and Q3 quartiles for that treatment group. Subject genotypes are indicted as GG or AA. 776 and 778 indicate GSK'776 (GSK2794776A an inactive diastereomer) or GSK'778 (GSK2794778A an RORgt inverse agonist)). Samples cultured in T cell maintenance medium are labelled no), while samples cultured in Th17 driving medium are labelled TH17.*

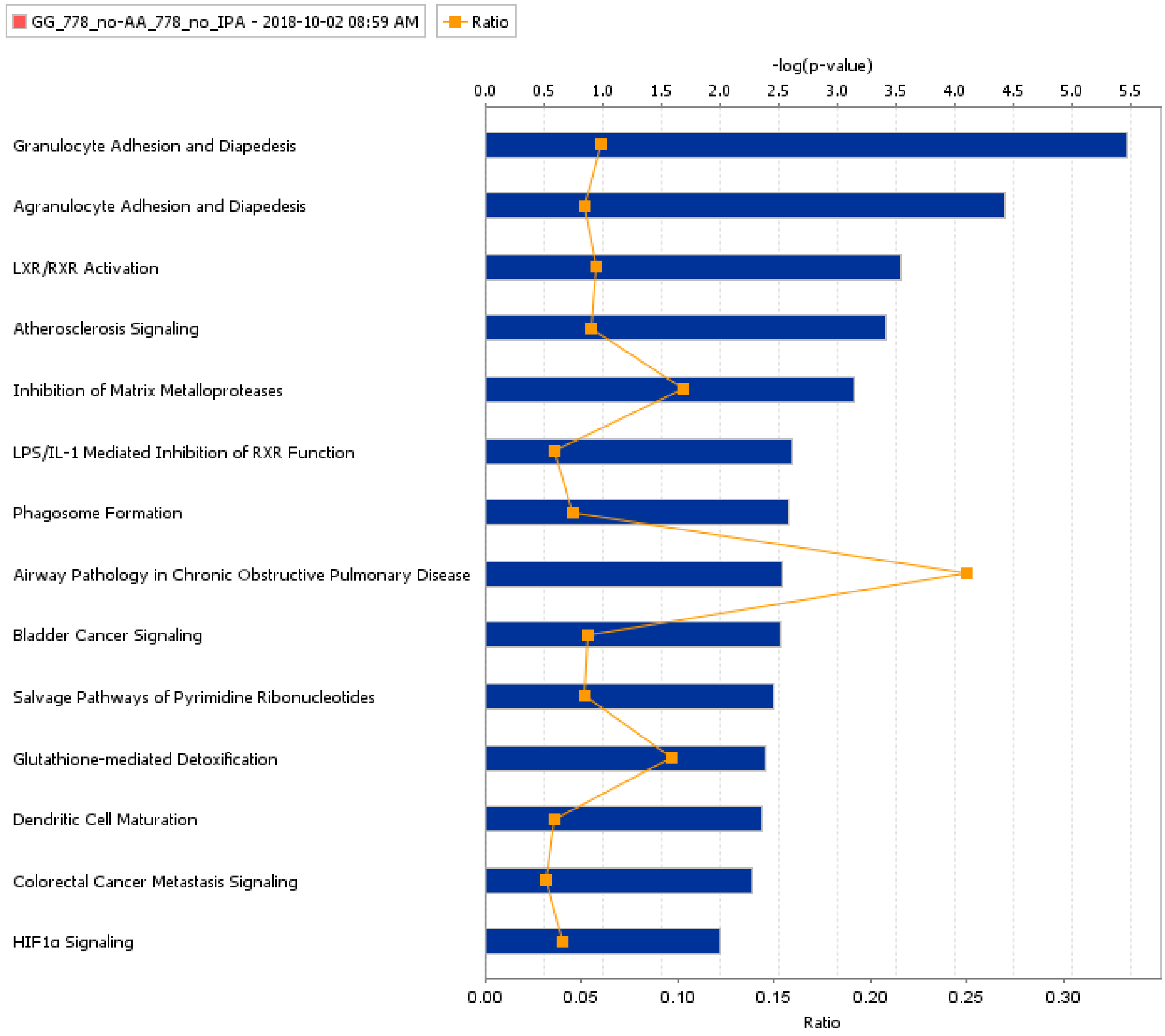

© 2000-2020 QIAGEN. All rights reserved.

Figure 7: *Canonical Pathway enrichment analysis: Ingenuity pathway enrichment analysis was completed using those gene transcripts reported as significantly differentially expressed when cells isolated form GG genotype subjects cultured for six days in T cell maintenance medium in the presence of an RORC inverse agonist where contrasted with cells isolated form AA genotype subjects and subjected to identical culture conditions. Enriched pathways were identified and ranked using a Fisher's Exact Test. The blue bars represent the -log(p-value) of these tests (i.e. larger bar size equates to statistical significance of the enrichment). The ratio of the number of differentially expressed genes relative to the number of genes included in the pathway is summarised by the orange line. Subject genotypes are indicted as GG or AA. While 776 and 778 indicate GSK'776 (GSK2794776A an inactive diastereomer) or GSK'778 (GSK2794778A an RORgt inverse agonist)). No indicates that samples cultured in T cell maintenance medium.*

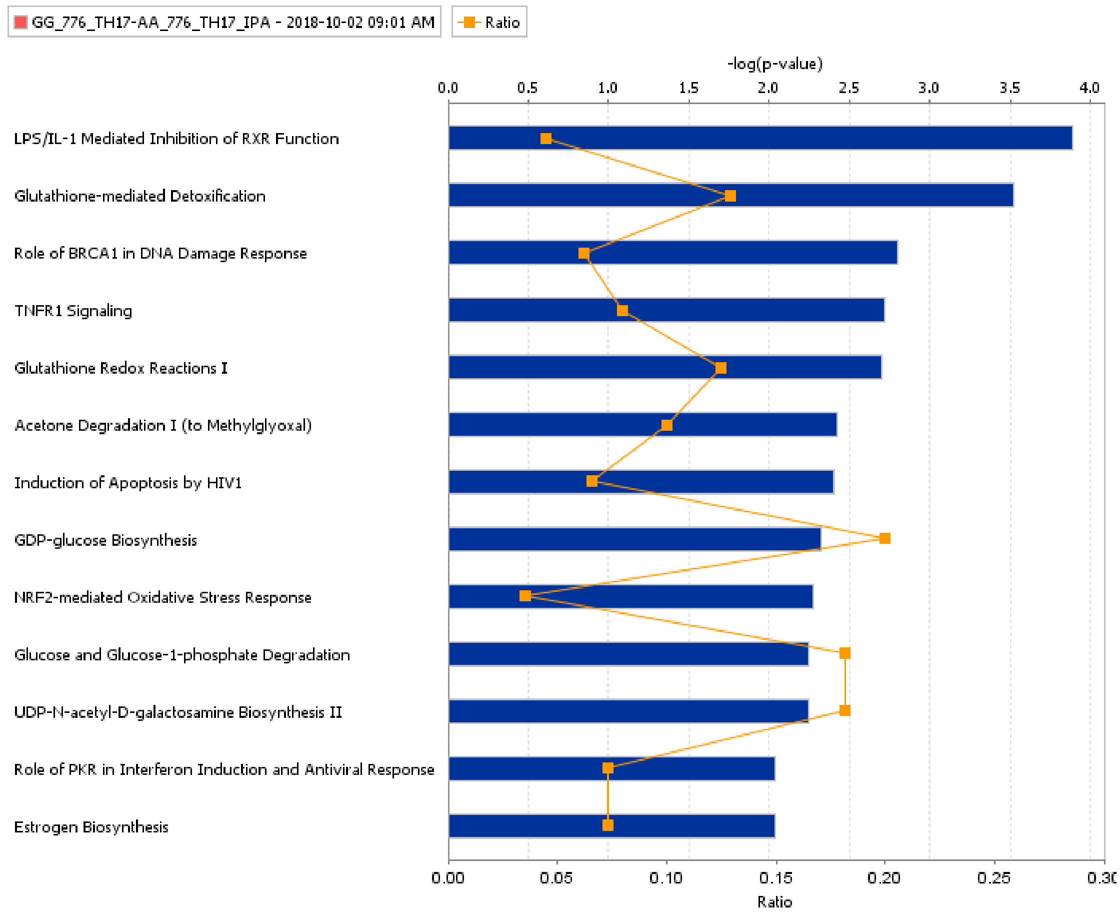

© 2000-2020 QIAGEN. All rights reserved.

Figure 8: *Canonical Pathway enrichment analysis: Ingenuity pathway enrichment analysis was completed using those gene transcripts reported as significantly differentially expressed when cells isolated form GG genotype subjects cultured for six days in Th17 driving medium in the presence of an inactive diastereomer where contrasted with cells isolated from AA genotype subjects and subjected to identical culture conditions. Enriched pathways were identified and ranked using a Fisher's Exact Test. The blue bars represent the -log(p-value) of these tests (i.e. larger bar size equates to statistical significance of the enrichment). The ratio of the number of differentially expressed genes relative to the number of genes included in the pathway is summarised by the orange line. Subject genotypes are indicted as GG or AA. While 776 and 778 indicate GSK'776 (GSK2794776A an inactive diastereomer) or GSK'778 (GSK2794778A an RORgt inverse agonist)). TH17 indicates that samples were cultured in Th17 driving medium.*

Ensembl Gene Identifier	Gene Symbol	Description	Change in Expression
ENSG00000074370	ATP2A3	ATPase sarcoplasmic/endoplasmic reticulum Ca2+ transporting 3(ATP2A3)	upregulated
ENSG00000131686	CA6	carbonic anhydrase 6(CA6)	upregulated
ENSG00000158485	CD1B	CD1b molecule(CD1B)	upregulated
ENSG00000106603	COA1	cytochrome c oxidase assembly factor 1 homolog(COA1)	upregulated
ENSG00000164935	DCSTAMP	dendrocyte expressed seven transmembrane protein(DCSTAMP)	upregulated
ENSG00000104936	DMPK	dystrophia myotonica protein kinase(DMPK)	upregulated
ENSG00000188820	CALHM6	Calcium homeostasis modulator family member 6	upregulated
ENSG00000132436	FIGNL1	fidgetin like 1(FIGNL1)	upregulated
ENSG00000134202	GSTM3	glutathione S-transferase mu 3(GSTM3)	upregulated
ENSG00000160255	ITGB2	integrin subunit beta 2(ITGB2)	upregulated
ENSG00000229921	KIF25-AS1	KIF25 antisense RNA 1(KIF25-AS1)	upregulated
ENSG00000100079	LGALS2	galectin 2(LGALS2)	upregulated
ENSG00000038945	MSR1	macrophage scavenger receptor 1(MSR1)	upregulated
ENSG00000271425	NBPF10	neuroblastoma breakpoint family member 10(NBPF10)	upregulated
ENSG00000149635	OCSTAMP	osteoclast stimulatory transmembrane protein(OCSTAMP)	upregulated
ENSG00000127948	POR	Cytochrome p450 oxidoreductase	upregulated
ENSG00000006747	SCIN	scinderin(SCIN)	upregulated
ENSG00000136436	CALCOCO2	calcium binding and coiled-coil domain 2(CALCOCO2)	downregulated
ENSG00000145384	FABP2	fatty acid binding protein 2(FABP2)	downregulated
ENSG00000214967	NPIPA7	nuclear pore complex interacting protein family member A7(NPIPA7)	downregulated
ENSG00000087157	PGS1	phosphatidylglycerophosphate synthase 1(PGS1)	downregulated
ENSG00000171772	SYCE1	synaptonemal complex central element protein 1(SYCE1)	downregulated

Table 2: *Summary details of 22 genes reported as significantly differentially expressed in all for statistical contrasts (see methods section of the main manuscript for details of the statistical model used). The "Change in Expression" column indicates the differential response observed in the GG subjects relative to the AA subjects.*
